# Disparities in computed tomography utilization for pediatric blunt trauma: a systematic review and meta-analysis comparing pediatric and non-pediatric trauma centers

**DOI:** 10.1007/s10140-023-02172-3

**Published:** 2023-09-23

**Authors:** Amir Hassankhani, Parya Valizadeh, Melika Amoukhteh, Payam Jannatdoust, Nikoo Saeedi, Paniz Sabeghi, Delaram J. Ghadimi, Jennifer H. Johnston, Ali Gholamrezanezhad

**Affiliations:** 1 Department of Radiology, Keck School of Medicine, University of Southern California (USC), 1441 Eastlake Ave Ste 2315, Los Angeles, CA 90089 USA; 2 Department of Radiology, Mayo Clinic, Rochester, MN USA; 3 School of Medicine, Tehran University of Medical Sciences, Tehran, Iran; 4 Student Research Committee, Islamic Azad University, Mashhad Branch, Mashhad, Iran; 5 School of Medicine, Shahid Beheshti University of Medical Sciences, Tehran, Iran; 6 Department of Diagnostic and Interventional Imaging, McGovern Medical School, University of Texas Health Science Center at Houston, Houston, TX USA

**Keywords:** Computed tomography, Pediatrics, Trauma center

## Abstract

Pediatric blunt trauma is a major cause of morbidity and mortality, and computed tomography (CT) imaging is vital for accurate evaluation and management. Pediatric trauma centers (PTCs) have selective CT practices, while non-PTCs may differ, resulting in potential variations in CT utilization. The objective of this study is to delineate disparities in CT utilization for pediatric blunt trauma patients between PTCs and non-PTCs. A systematic review and meta-analysis were conducted following established guidelines, searching PubMed, Scopus, and Web of Science up to March 3, 2023. All studies examining CT utilization in the management of pediatric (aged < 21 years) blunt trauma and specifying the type of trauma center(s) were included, and data were extracted and analyzed using STATA software version 17.0. An analysis of 30 studies revealed significant variations in CT scan utilization among pediatric blunt trauma patients across different types of trauma centers. PTCs exhibited lower pooled rates of abdominopelvic CT scans (35.4% vs. 44.9%, p < 0.01), cranial CT scans (36.9% vs. 42.9%, p < 0.01), chest CT scans (14.5% vs. 25.4%, p < 0.01), and cervical spine CT scans (23% vs. 45%, p < 0.01) compared to adult or mixed trauma centers (ATCs/MTCs). PTCs had a pooled rate of 54% for receiving at least one CT scan, while ATCs/MTCs had a higher rate of 69.3% (p < 0.05). The studies demonstrated considerable heterogeneity. These findings underscore the need to conduct further research to understand the reasons for the observed variations and to promote appropriate imaging usage, minimize radiation exposure, and encourage collaboration between pediatric and adult trauma centers.

**Supplementary Information:**

The online version contains supplementary material available at 10.1007/s10140-023-02172-3.

## Introduction

Blunt trauma represents a significant source of morbidity and mortality among pediatric patients, resulting in a substantial number of emergency department visits and hospital admissions. The timely and accurate evaluation of pediatric blunt trauma is crucial for effective management and the prevention of potential complications [1–3]. Computed tomography (CT) imaging plays a pivotal role in the diagnostic process, providing detailed anatomical information and aiding in the identification of injuries [4–6].

Pediatric trauma centers (PTCs) are dedicated facilities designed to provide comprehensive care for children and adolescents with traumatic injuries. These specialized centers offer a range of resources, expertise, and protocols tailored specifically to the unique needs of pediatric patients [7–9]. PTCs employ a more selective approach to CT imaging, taking into account alternative modalities and utilizing lower-dose CT protocols to minimize radiation exposure while maintaining diagnostic accuracy [10–12]. However, several factors, including geographical limitations, resource availability, and system-level considerations, may result in pediatric patients with blunt trauma initially being assessed at non-PTCs. These non-PTCs encompass a broader spectrum of trauma centers that cater to both adult and pediatric populations, and their imaging practices may differ from those of dedicated pediatric centers. Recent studies have indicated the potential for variations in the utilization of CT imaging in the evaluation of pediatric blunt trauma between PTCs and non-PTCs [1–3].

To optimize the diagnostic approach for pediatric patients with blunt trauma, it is crucial to comprehend the disparities in CT utilization between PTCs and non-PTCs. Consequently, we undertook a systematic review and meta-analysis to investigate this subject comprehensively.

## Methods

Following the guidelines outlined in the Preferred Reporting Items for Systematic Review and Meta-Analyses (PRISMA) statement [13], a comprehensive literature search was conducted on March 3, 2023. The search encompassed three major databases: PubMed, Scopus, and Web of Science. To ensure a thorough search, carefully crafted search terms specific to each database were employed, including (“pediatric*” OR “paediatric*” OR “child*” OR “neonat*” OR “infant*” OR “toddler*” OR “preschool” OR “pre-school” OR “juvenile” OR “young adult*”) AND (“tomography, x-ray computed” OR “CT scan” OR “CT-scan” OR “computed tomography” OR “computerized tomography”) AND (“trauma center*” OR “pediatric trauma center*” OR “adult trauma center*” OR “trauma unit*” OR “pediatric trauma unit” OR “adult trauma unit*” OR “ED” OR “emergency department” OR “accident and emergency” OR “A&E” OR “emergency room”) AND (“wounds and injuries” OR “wounds, nonpenetrating” OR “blunt trauma” OR “blunt injury” OR “nonpenetrating trauma” OR “non-penetrating trauma” OR “nonpenetrating injury” OR “non-penetrating injury”). To ensure comprehensive coverage of relevant studies, a thorough manual search of references from the included studies was conducted. Deduplication, screening, and data extraction processes were carried out using the AutoLit platform, developed by Nested Knowledge in St. Paul, Minnesota.

All studies examining the utilization of CT scans in the management of pediatric (aged < 21 years) blunt trauma and specifying the type of trauma center(s) were included in this review. No restrictions were placed on the date, country of origin, or study design. Exclusions consisted of non-English literature, conference abstracts, editorial comments, author responses, case reports, case series with fewer than 10 eligible patients, review articles, and irrelevant papers. Two authors evaluated the title, abstract, and/or full text of each article, independently, resolving any uncertainties or ambiguities through consultation with a senior coauthor.

A comprehensive set of relevant details from each eligible paper was extracted, including the first author's name, publication year, country of origin, study design, trauma center type and level, study aim, sample size, patients' characteristics and outcomes, as well as CT types and rates.

To assess the quality of the studies included in our analysis, we utilized the Joanna Briggs Institute (JBI) critical appraisal tool for analytic cross-sectional studies [14]. This tool encompasses various domains that evaluate different aspects of study quality, such as objectives and research questions, study design, sampling strategy, data collection, data analysis, ethics considerations, results and findings, and conclusion and implications. For each criterion, researchers can choose from response options like "Yes," "No," "Unclear," or "Not Applicable." Through this systematic evaluation, we aimed to ensure the reliability and rigor of the studies, establishing the validity of our conclusions.

### Statistical analysis

To address the varying data formats in the included studies, where injury severity score (ISS) and age data were often presented as medians and interquartile ranges, we utilized an online tool developed based on the methods proposed by Luo et al., Wan et al., and Shi et al. This tool enabled us to convert medians to means, facilitating comparisons across studies [15–17].

Given the high methodological heterogeneity observed among the studies, we used a random effects model for all meta-analyses in this study. Our primary objective was to compare the rates of CT scan utilization between PTCs and non-PTCs. To achieve this, we conducted subgroup meta-analyses, classifying the studies into two groups: PTC and adult or mixed trauma centers (ATC/MTC).

To assess overall heterogeneity across the studies, we employed the I^2^ statistic [18]. If the I^2^ value exceeded 50%, indicating substantial heterogeneity, we performed univariable meta-regressions with trauma center type and level as covariates. If the meta-regression analysis yielded significant results for the effect of trauma center level, we conducted a subgroup meta-analysis, stratifying the studies based on trauma center level.

In addition, when available, we performed univariable meta-regression analyses using the mean ISS and mean age of the study sample as covariates. We also examined the potential impact of publication year on the reported rates by conducting univariable meta-regression with publication year as a covariate. For significant continuous variables, we organized the studies in the forest plot based on the significant variable to assess the trend between that variable and CT rates visually.

If a sufficient number of studies were included, we conducted bivariable meta-analyses with mean ISS and mean age as covariates in separate models. The results of the meta-regression analyses were assessed using chi-square tests to evaluate the heterogeneity explained by the model and alterations in the Bayesian information criterion (delta-BIC) as a measure of heterogeneity explained in the reduced model.

We also performed a comparative meta-analysis to calculate the pooled odds ratio (OR) of undergoing each type of CT scan in ATC/MTC patients compared to blunt trauma patients in PTCs. For this OR meta-analysis, we used a random effects Restricted Maximum Likelihood (REML) model. To evaluate publication bias of OR meta-analysis, we used funnel plots and conducted nonparametric trim-and-fill analysis if visual asymmetry was observed. Since the classic funnel plot method is limited for assessing publication bias for proportion data [19], we assessed publication bias of proportion meta-analyses using Doi plots [20]. Effect sizes were transformed using the Freeman-Tukey transformation for this analysis [21].

All statistical analyses were performed using STATA software (Version 17.0, Stata Corp, College Station, TX). We utilized the "metapreg," "metaprop_one" [22], and "LFK" user-made packages for the analyses [23], which provided the necessary functionality for conducting the meta-analyses and performing statistical assessments.

## Results

### Screening and selection of articles

**Fig. 1:**
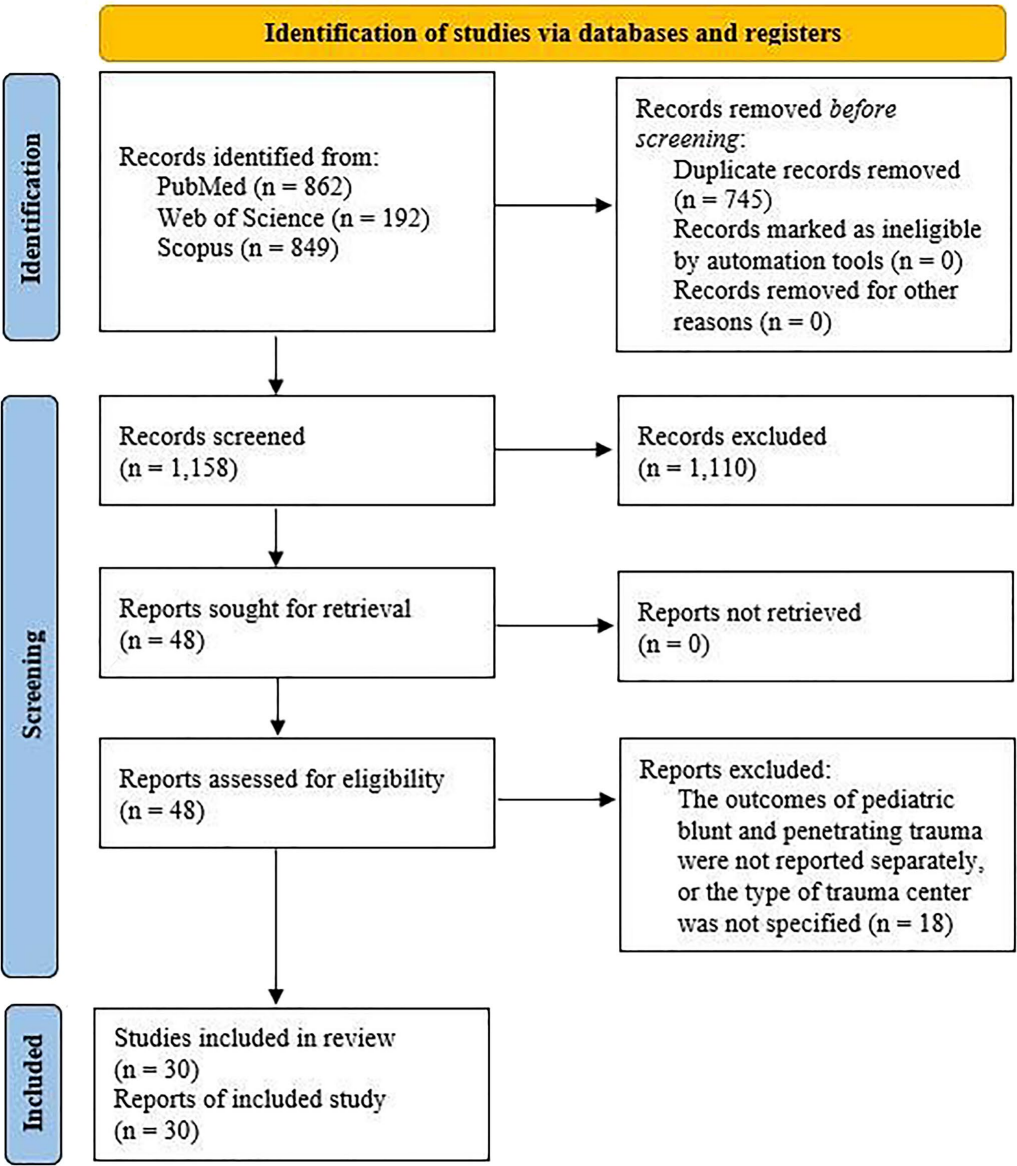
PRISMA flow diagram showing the review process. PRISMA: Preferred Reporting Items for Systematic Reviews and Meta-Analyses

During our systematic literature search, a total of 1,903 articles was identified using a predefined search strategy. Following the removal of duplicate records, we screened 1,158 papers based on their title and abstract. This led to the exclusion of 1,110 articles, comprising 78 conference abstracts, 5 editorial comments, 100 case reports, 49 review articles, 176 original papers not involving pediatric populations, 84 original papers not focused on pediatric blunt trauma, and 618 papers unrelated to the topic of interest. Subsequently, the full text of the remaining 48 papers was retrieved and reviewed. After evaluation, 18 articles were excluded because they did not specify the type of trauma center(s) or any of the outcomes of interest. Ultimately, 30 articles that met the inclusion criteria were identified and incorporated into our analysis. The entire screening process and application of eligibility criteria were summarized using a flow diagram in accordance with PRISMA guidelines (Fig. 1).

### Study and participants characteristics

Table 1 provides a summary of the characteristics of included articles. Among the 30 studies examined, 17 reported CT rates in PTCs, 8 reported CT rates in non-PTCs, and 5 provided a comparison of CT rates between PTCs and non-PTCs. These studies were conducted in various countries, including 24 studies from the United States, three from Canada, one from the Netherlands, one from Australia, and one from Turkey. The study designs varied, with 23 retrospective cohorts, 5 prospective cohorts, 1 randomized controlled trial, and 1 case–control study. Trauma center types and levels also differed across the studies, with the majority conducted in level 1 trauma centers (N = 24). The aims of the studies encompassed evaluating CT rates for different types of traumas (N = 10), comparing CT rates among different trauma center types (N = 5),

**Table 1:** Characteristics of the included studies

Author, Year of publication	Country	Study design	Trauma center type and level	Study aim	Included patients	Type of CT scan utilized
Wiitala et al., 2022 [1]	United States	Case–control matched for age, sex, race, and Glasgow Coma Scale	PL1/PL2-AL1-AL2	Propensity matched comparison of CT scan rate between PL1 vs PL2-AL1-AL2	All abdominal or thoracic blunt trauma with ISS > 15 (severe)	Chest, head, abdomen, other
Walther et al., 2016 [2]	United States	Retrospective cohort	PL1/AL1	Comparison of CT scan rate between AL1 and PL1	Adolescents with severe blunt trauma in PL1 and AL1 with LOS > 1	Chest, head, abdomen
Walther et al., 2014 [3]	United States	Retrospective cohort	PL1-PL2/AL1	Comparison of CT scan rate between PL1, PL2, and AL1	Adolescents with blunt trauma in PL1 with LOS > 1 in Ohio	Head, abdomen
Brinke et al., 2021 [4]	Netherlands	Retrospective cohort	ML2	Assessing the potential effect of developing a new protocol on CT scan rate	Blunt trauma with suspected cervical spine injury	Cervical spine
Streck et al., 2012 [6]	United States	Retrospective cohort	PL1	Evaluating a scoring system to predict intra-abdominal injury	All alert blunt trauma cases younger than 16	Abdomen
Stephens et al., 2017 [24]	United States	Retrospective cohort	PL1	Assessing the potential effect of developing a new protocol on CT scan rate	Blunt trauma patients who received a CXR	Chest
Sharma et al., 2022 [25]	United States	Retrospective cohort	AL1	Reviewing cervical spine CT scan rate in an AL1 center	Blunt trauma	Cervical spine
Schonfeld et al., 2013 [26]	United States	Prospective cohort	PL1	Reviewing head CT scan rate in a PL1 center	Minor blunt head trauma	Head, abdomen
Sathya et al., 2018 [10]	United States	Retrospective cohort	PL1- PL2/AL1-AL2-ML1-ML2	Comparison of CT scan rate between PL1, PL2 and AL1-AL2-ML1-ML2	All blunt trauma with AIS > 1	chest, head, abdomen
Plackett et al., 2015 [27]	United States	Retrospective cohort	ML1	Reviewing head CT scan rate in a ML1 center	All blunt trauma patients	head
Phillips et al., 2021 [11]	Australia	Prospective cohort	PL1	Reviewing cervical spine CT scan rate in a PL1 center in patients with suspected injury and assessing the potential effect of developing a new guideline on CT scan rate	Blunt trauma with suspected cervical spine injury	Cervical spine
Pariaszevski et al., 2022 [28]	United States	Retrospective cohort	PL1/ML1	Evaluating the trends in CT scan rate after separation of adult and pediatric trauma centers and comparing CT scan rate between PL1 and ML1	All blunt trauma cases	Chest, head, abdomen, other
Odia et al., 2020 [29]	United States	Retrospective cohort	PL1	Assessing the potential effect of developing a new protocol on CT scan rate	Hyponymically stable blunt abdominal trauma cases	Abdomen
Nigrovic et al., 2015 [30]	United States	Prospective cohort	PL1	Assessing the potential effect of efforts on reducing cranial CT scan rate	Minor blunt head trauma cases	Head
Moore et al., 2013 [31]	United States	Retrospective cohort	PL2	Reviewing CT scan rates in a PL2 center	All blunt trauma cases	Chest, head, abdomen, other
McGrew et al., 2018 [32]	United States	Retrospective cohort	PL2	Assessing the potential effect of developing a new guideline on CT scan rate	All blunt trauma cases	Chest, head, abdomen, other
Mahdi et al., 2023 [33]	United States	Prospective cohort	PL1	Assessing the potential effect of developing a new guideline on CT scan rate	All blunt trauma cases	Chest, head, abdomen, other
Schonenberg Llach et al., 2021 [34]	United States	Retrospective cohort	ML1	Assessing the potential effect of a new pathway on CT scan rate	All blunt trauma cases	Cervical spine
Livingston et al., 2013 [35]	Canada	Retrospective cohort	ML1	Reviewing CT scan rate and radiation dose in a ML1 center	All blunt trauma cases with ISS > 12	Chest, head, abdomen, cervical spine
Kuas et al., 2022 [36]	Turkey	Prospective cohort	ML1	Evaluating the role of laboratory markers in predicting abdominal solid organ injury	Blunt abdominal trauma cases	Abdomen
Kolousek et al., 2023 [37]	United States	Retrospective cohort	AL1	Reviewing CT scan rate in an AL1 center	All blunt trauma cases	Abdomen
Kim et al., 2005 [38]	United States	Retrospective cohort	PL1	Reviewing CT scan rate and radiation dose in a PL1 center	All trauma cases	Chest, head, abdomen, cervical spine
Holmes et al., 2017 [39]	United States	Randomized controlled trial	PL1	Assessing the potential effect of implementing abdominal ultrasound on CT scan rate	hyponymically stable blunt abdominal trauma cases	Abdomen
Haasz et al., 2015 [40]	Canada	Retrospective cohort	PL1	Developing a tool for predict pelvic fractures	Blunt trauma patients with suspected pelvic fracture	Pelvic
Golden et al., 2016 [41]	United States	Retrospective cohort	PL1	Reviewing CT scan rate in a PL1 center	All blunt trauma patients with a chest x-ray in admission	Chest
Gaffley et al., 2020 [42]	United States	Retrospective cohort	PL1	Assessing the potential effect of developing a new guideline on CT scan rate	All blunt trauma cases	All
Edwards et al., 2021 [43]	United States	Retrospective cohort	PL1	Reviewing CT scan rate in an PL1 center	All blunt trauma cases	All
Downie et al., 2023 [44]	United States	Retrospective cohort	ML1	Assessing the potential effect of developing a new protocol on CT scan rate	Blunt trauma cases with a chest x-ray	Chest
Brinkman et al., 2015 [45]	United States	Retrospective cohort	PL1	Reviewing CT scan rates in an PL1 center and comparing CT scan rate and radiation dose between a PL1 center and a referring facility	Blunt trauma cases	Chest, head, abdomen
Beno et al., 2022 [46]	Canada	Retrospective cohort	PL1	Assessing the potential effect of efforts on reducing abdominopelvic CT rate	Blunt trauma cases with low risk for abdominal injury	Abdomen

*AL1&2* Adult Level 1 & 2 trauma centers, *ISS* Injury Severity Score, *LOS* Length of Hospital Stay, *ML1&2* Mixed Level 1 and 2 trauma centers, *PL1&2* Pediatric Level 1 & 2 trauma centers

developing prediction tools for specific injuries (N = 3), and assessing the effects of interventions and new protocols on CT rates (N = 12). Among the included studies, abdominopelvic CT rates were reported in the majority (N = 19), while rates for head (N = 14), chest (N = 12), cervical (N = 10), and other regions (N = 3) CT scans were less commonly reported.

Table 2 presents a comprehensive summary of the included studies focusing on pediatric patients with blunt trauma and their utilization of CT scans. The table provides information such as the number of patients, gender distribution, age, and ISS for each study. It also displays the proportions of patients who underwent CT scans for specific body regions. Additionally, the table includes reported outcomes such as the admission rate to intensive care units and the mortality rate. In cases where studies differentiated between PTCs and non-PTCs, this distinction is indicated in the table.

**Table 2:** Characteristic of pediatric blunt trauma patients and utilization of CT scans in included studies

Author, Year of publication	Number of included patients			Male (%)	Age (years)	ISS	LOS	Outcomes		CT rates (%)					
	All	PTC	ATC/MTC					ICU rate (%)	Mortality rate (%)	Overall	Head	Chest	Abdominopelvic	Cervical	Other
Wiitala et al., 2022 [1]	2790	1395	ATC: 1395	PTC: 62.2 non-PTC: 60.6	9, (4–14) Median, (IQR)	PTC: 20, (17–26) non-PTC: 21, (17–26) Median, (IQR)	PTC: 4, (2–8) non-PTC: 4, (2–8) Median, (IQR)	NS	PTC: 3.9 non-PTC: 5.1	NS	PTC: 66.3 non-PTC: 66.3	PTC: 21.9 non-PTC: 34.7	PTC: 39.6 non-PTC: 45.5	NA	PTC: 54.1 non-PTC: 48
Walther et al., 2016 [2]	11,453	5588	ATC: 5865	PTC: 79.2 non-PTC: 77.5	PTC: 17.4 ± 1.3 non-PTC: 17.5 ± 1.3 Mean ± SD	33, (29–38) Median, (IQR)	PTC: 10, (5–20) non-PTC: 10, (6–20) Median, (IQR)	NS	PTC: 8.3 non-PTC: 8.6	NS	PTC: 27.9 non-PTC: 40.5	PTC: 22.1 non-PTC: 28	PTC: 29.4 non-PTC: 43.1	NA	NA
Walther et al., 2014 [3]	4857	1680	ATC: 3177	PTC: 71.6 non-PTC: 66.3	PTC: 15.9 ± 0.9 non-PTC: 17.6 ± 1.2 Mean ± SD	PTC: 5, (4–9) non-PTC: 9, (4–14) Median, (IQR)	NS	NS	PTC: 0.6 non-PTC: 1.7	NS	PTC: 14.2 non-PTC: 32.3	NA	PTC: 17.2 non-PTC: 24.6	NA	NA
Brinke et al., 2021 [4]	253	0	MTC: 253	53.4	10.4 Mean	4.6 Mean	2.2 Mean	NS	NS	NS	NA	NA	NA	26.9	NA
Streck et al., 2012 [6]	125	125	0	NS	8.51 ± 4.97 Mean ± SD	13.6 ± 10 Mean ± SD	5.8 Mean	NS	4.8	NA	NA	NA	77.6	NA	NA
Stephens et al., 2017 [24]	2951	2951	0	65.0	13, (6–16) Median, (IQR)	9, (4–17) Median, (IQR)	NS	NS	2.0	NA	NA	50.8	NA	NA	NA
Sharma et al., 2022 [25]	546	0	ATC: 546	68.2 (with CT group)	16 Mean (with CT group)	NS	NS	NS	NS	NA	NA	NA	NA	61.7	NA
Schonfeld et al., 2013 [26]	1381	1381	0	60.3	NS	NS	NS	NS	NS	NA	19.6	NA	NA	NA	NA
Sathya et al., 2018 [10]	59,010	16,573	ATC: 22,695 MTC: 19,742	NS	NS	NS	NS	NS	NS	PTC: 43.4 ATC: 55.8 MTC: 56.8	PTC: 35.6 ATC: 48.7 MTC: 48.2	PTC: 3.3 ATC: 22.5 MTC: 23.1	PTC: 13.4 ATC: 31.5 MTC: 31.3	NA	NA
Plackett et al., 2015 [27]	2867	2867	0	48.7 (with CT group)	8, (2–13) Median, (IQR) (with CT group)	5, (2–6) Median, (IQR) (with CT group)	2.4 ± 2.5 Mean ± SD (with CT group)	NS	NS	NA	22.0	NA	NA	NA	NA
Phillips et al., 2021 [11]	973	973	0	66.1	10.9, (7.1, 13.6), (0.01–15.99) Median, (IQR), (Range)	NS	NS	2.8	0.4	NA	NA	NA	NA	13.4	NA
Pariaszevski et al., 2022 [28]	463	235	MTC: 228	PTC: 66.0 non-PTC: 63.6	NS	PTC: 3 non-PTC: 5 Mean	NS	NS	NS	PTC: 55.3 non-PTC: 63.6	PTC: 41.3 non-PTC: 52.2	PTC: 8.1 non-PTC: 16.7	PTC: 13.2 non-PTC: 20.2	PTC: 28.9 non-PTC: 41.7	PTC: 25.5 non-PTC: 44.7
Odia et al., 2020 [29]	115	115	0	59.1	8, (5–12) Median, (IQR)	4, (1–6) Median, (IQR)	NS	NS	NS	NA	NA	NA	60.0	NA	NA
Nigrovic et al., 2015 [30]	6856	6856	0	60.4	NS	NS	NS	NS	NS	NA	15.2	NA	NA	NA	NA
Moore et al., 2013 [31]	174	174	0	63.2	7 ± 5 Mean ± SD	10 ± 10 Mean ± SD	NS	NS	4.0	87.9	74.7	32.2	60.3	44.8	NA
McGrew et al., 2018 [32]	1934	1934	0	NS	7.5 ± 4.8 Mean ± SD	5, (4–10) Median, (IQR)	2.8 Mean	21.3	1.2	49.5	50.2	14.6	34.0	NA	NA
Mahdi et al., 2023 [33]	424	424	0	63.4	8.66 ± 4.75 Mean ± SD	7.7 ± 8.8 Mean ± SD	3.6 ± 5.8 Mean ± SD	NS	NS	NA	60.4	22.4	40.3	43.9	NA
Schonenberg Llach et al., 2021 [34]	358	0	MTC: 358	61.7	8.1 ± 4.4 Mean ± SD	1.5, (1–8) Median, (IQR)	NS	NS	NS	NA	NA	NA	NA	31.8	NA
Livingston et al., 2013 [35]	153	NS	MTC: 157	73.2	9.6 ± 6.1 Mean ± SD	22.5 ± 9.6 Mean ± SD	10 Mean	NS	9.6	84.7	NA	NA	NA	NA	NA
Kuas et al., 2022 [36]	323	NR	MTC: 323	36.5	10, (7–15) Median, (IQR)	NS	NS	NS	NS	NA	NA	NA	87.6	NA	NA
Kolousek et al., 2023 [37]	546	0	ATC: 546	67.0 (with CT group)	16.6 Mean (with CT group)	17.1 ± 11.7 Mean ± SD (with CT group)	NS	NS	3.1	NA	NA	NA	59.3	NA	NA
Kim et al., 2005 [38]	506	506	0	NS	14.49 ± 16.22 Mean ± SD	7.91 ± 7.89 Mean ± SD	NS	NS	NS	62.1	55.3	NA	28.1	21.7	8.3
Holmes et al., 2017 [39]	925	925	0	64.3	9.7 ± 5.3	NS	NS	16.4	NS	53.5	NA	NA	53.5	1.7	NA
Haasz et al., 2015 [40]	1121	1121	0	63.0	9, (2–17) Median, (Range)	NS	NS	NS	2.3	NA	NA		48.7	NA	NA
Golden et al., 2016 [41]	1035	1035	0	64.4	7.1 ± 4.7 Mean ± SD	NS	NS	NS	1.2	NA	NA	13.4	NA	NA	NA
Gaffley et al., 2020 [42]	998	998	0	62.8	NS	5, (2–9) Median, (IQR)	2, (1–3) Median, (IQR)	NS	NS	42.1	NA		42.1	NA	NA
Edwards et al., 2021 [43]	735	735	0	NS	6.9 ± 4.6 Mean ± SD	6.5 Mean	2.5 ± 7 Mean ± SD	NS	NS	57.8	45.7	23.1	26.9	34.8	NA
Downie et al., 2023 [44]	1056	1056	0	64.2	8, (1 day-17) Median, (Range)	(0–59) Range	NS	NS	NS	NA	NA	19.0	NA	NA	NA
Brinkman et al., 2015 [45]	321	321	0	63.9	10.5 ± 1.2 Mean ± SD	4, (2–9) Median, (IQR)	NS	24.0	NS	27.1	8.7	6.5	20.9	NA	NA
Beno et al., 2022 [46]	804	804	0	63.3	NS	NS	NS	NS	NS	NA	NA	NA	39.2	NA	NA

*ATC* Adult trauma center, *IQR* InterQuartile Range, *ISS* Injury Severity Score, *LOS* Length of Hospital Stay, *MTC* Mixed Trauma Center, *NA* Not Available, *NS* Not Specified, *PL1&2* PTC: Pediatric Trauma Center, *SD* Standard Deviation

### Quality assessment

Table 1 of Supplementary File presents a summary of the responses to each question included in the JBI critical appraisal tool for analytic cross-sectional studies. The results indicate that the majority of the included studies demonstrated acceptable methodological quality.

The table highlights specific studies excluded from the quantitative synthesis. These exclusions were guided by the studies' particular inclusion criteria, which centered on cases with a strong clinical suspicion of certain injury types, including those showing positive signs during physical examination. Excluding these studies aimed to ensure that the quantitative synthesis findings truly represented the wider population of pediatric blunt trauma patients, without being skewed by a subset of cases with specific clinical suspicions.

### Quantitative synthesis

#### Abdominopelvic CT scans

**Fig. 2:**
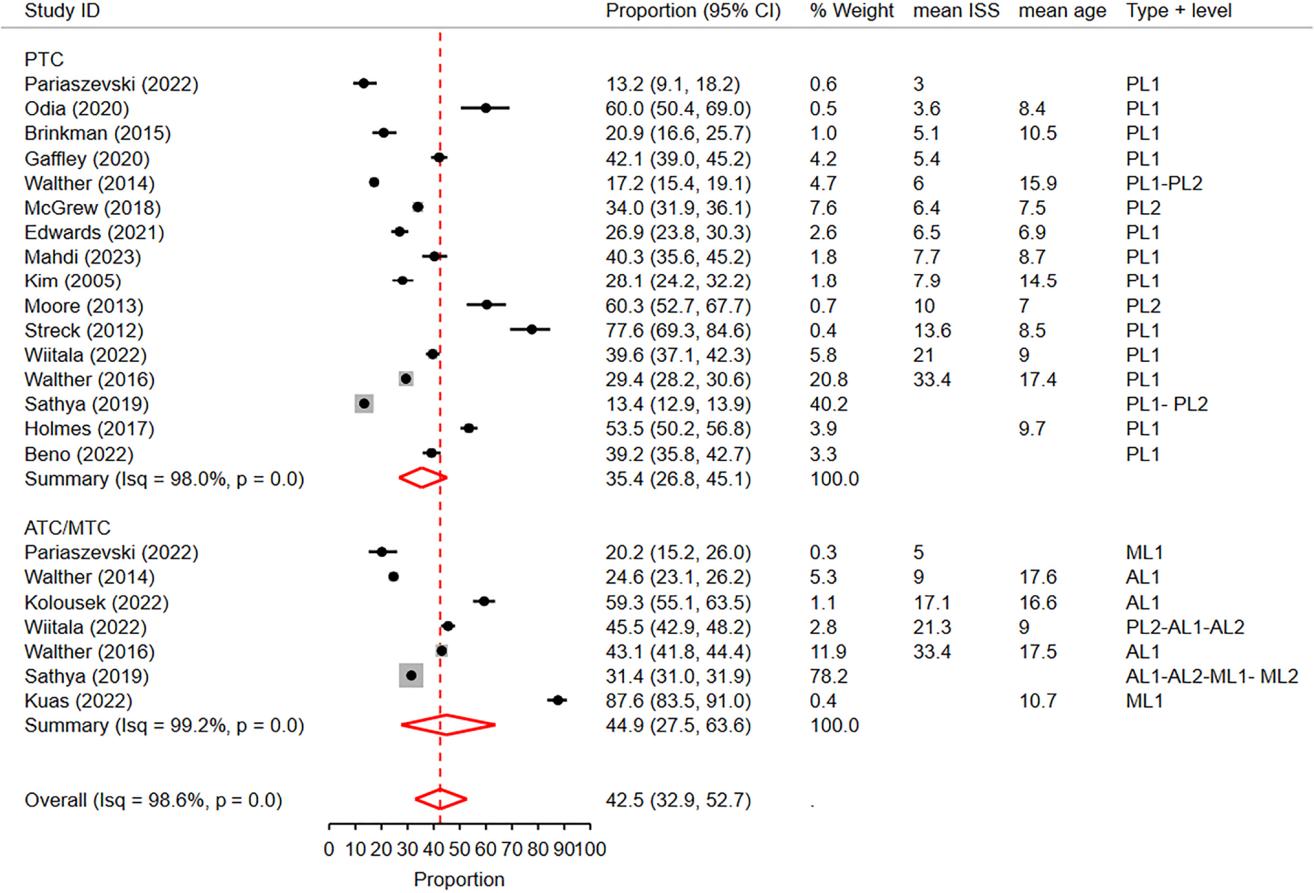
Forest plot of the random effects meta-analysis of the proportion of pediatric blunt trauma cases receiving abdominopelvic CT scans in pediatric trauma centers and adult/mixed trauma centers. The studies within each subgroup are arranged based on the mean Injury Severity Score. AL1&2: Adult Level 1 & 2 trauma centers. ATC: Adult trauma center. CI: Confidence Interval. ISS: Injury Severity Score. ML1&2: Mixed Level 1 and 2 trauma centers. MTC: Mixed Trauma Center. PL1&2: Pediatric Level 1 & 2 trauma centers. PTC: Pediatric Trauma Center

We included 18 unique studies in this analysis. Out of these, 16 studies reported rates in PTCs, while 7 studies assessed rates in ATCs/MTCs. The proportion meta-analysis revealed that the overall rate of receiving abdominopelvic CT scans among pediatric blunt trauma patients was 42.5% (95% CI: 32.9%—52.7%) (Fig. 2). There was a high level of heterogeneity among the studies (I^2^ = 98.6%).

Through univariate metaregression analysis, we found a significant effect of trauma center types (PTC vs. ATC/MTC) on the reported rates (p < 0.01, delta-BIC = 2248.5). The subgroup meta- analysis showed that PTCs had a significantly lower abdominopelvic CT rate, with a pooled rate of 35.4% (95% CI: 26.8%—45.1%), compared to 44.9% (95% CI: 27.5%—63.6%) in ATCs/MTCs (Fig. 2). On the other hand, a univariate metaregression based on trauma center level (Level 1 vs. Level 2 or mixed) did not demonstrate a statistically significant effect of trauma center level on the reported CT rates (p = 0.07).

Furthermore, in the univariable metaregression analysis, we found that mean age (p < 0.01, delta BIC = 40.4) and mean ISS scores (p < 0.01, delta BIC = 28.3) significantly affected the observed rates, while publication year did not have a significant effect (p = 0.7, delta BIC = -3.0). Upon visual inspection of forest plots sorted by mean ISS scores and mean age, a trend was observed indicating higher CT rates in higher ISS scores and younger ages (Figs. 1.1 and 1.2 of Supplementary File). It should be noted that mean ISS scores and mean age were reported in only a proportion of the included studies.

Subsequently, we conducted bivariate metaregression analyses to combine the observed significant effects. In the bivariable metaregression of trauma center type and mean ISS scores and mean age, we found that the effect of trauma center type remained significant even after controlling for mean ISS score (p < 0.01, delta BIC = 230.6) and mean age (p < 0.01, delta BIC = 218.9). Furthermore, the effects of mean age and mean ISS scores also remained significant in the bivariate model (p < 0.01, delta BIC = 42.3 and p < 0.01, delta BIC = 29, respectively).

**Fig. 3:**
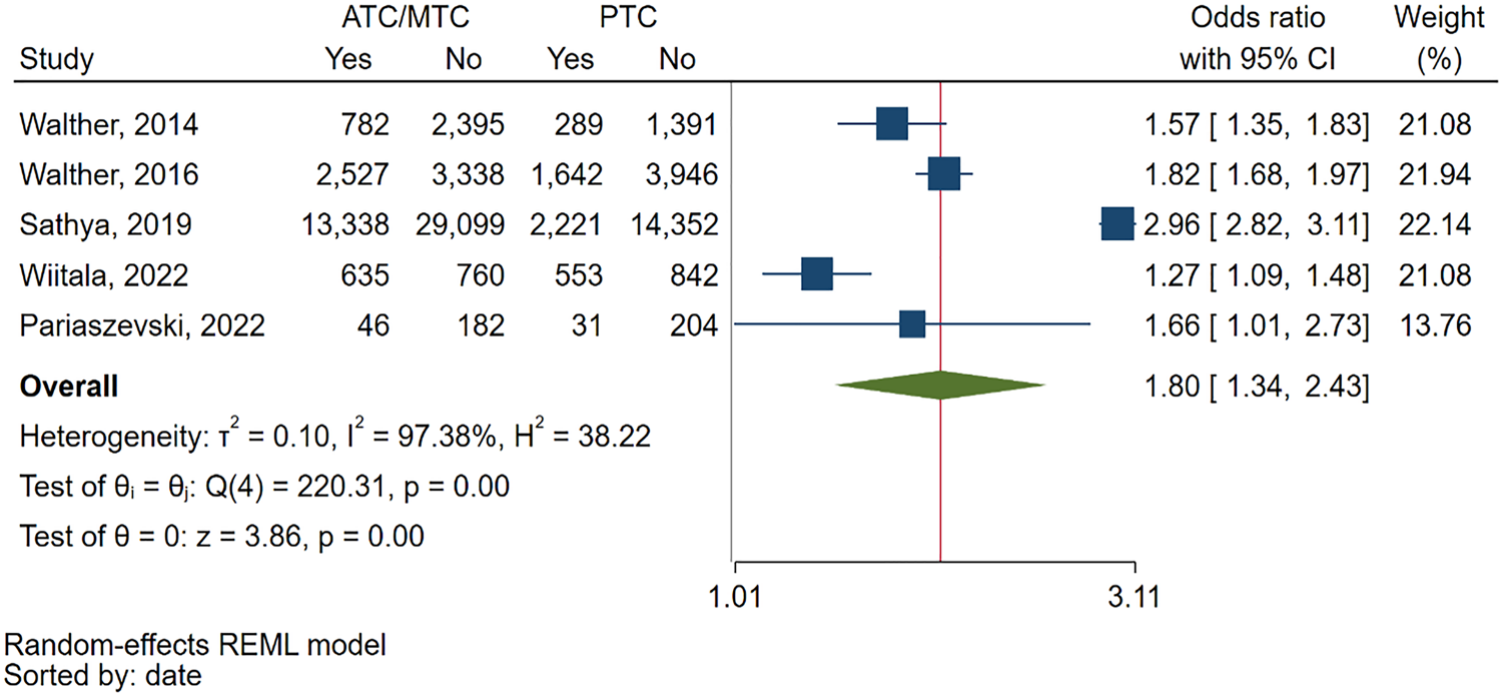
Forest plot of the random effects odds ratio meta-analysis of five studies that comparatively report the rates of abdominopelvic CT scans in pediatric blunt trauma patients in adult or mixed trauma centers compared to exclusively pediatric trauma centers. ATC: Adult Trauma Center. CI: Confidence Interval. MTC: Mixed Trauma Center. PTC: Pediatric Trauma Center

Among the studies included in our analysis, a total of 5 studies comparatively reported abdominopelvic CT rates in ATCs/MTCs compared to PTCs, allowing for an OR meta-analysis. The findings from the comparative meta-analysis revealed a significant difference, indicating that abdominopelvic CT scans are significantly more common in ATCs/MTCs compared to PTCs, with a pooled OR of 1.8 (95% CI: 1.34—2.43) (Fig. 3).

#### Cranial CT scans

**Fig. 4:**
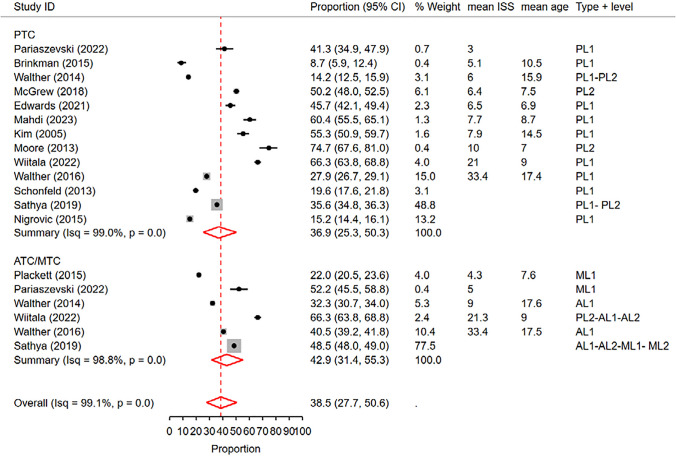
Forest plot of the random effects meta-analysis of the proportion of pediatric blunt trauma cases receiving cranial CT scans in pediatric trauma centers and adult/mixed trauma centers. The studies within each subgroup are arranged based on the mean Injury Severity Score. AL1&2: Adult Level 1 & 2 trauma centers. ATC: Adult trauma center. CI: Confidence Interval. ISS: Injury Severity Score. ML1&2: Mixed Level 1 and 2 trauma centers. MTC: Mixed Trauma Center. PL1&2: Pediatric Level 1 & 2 trauma centers. PTC: Pediatric Trauma Center

In this analysis, we included 13 studies conducted in PTCs and 6 studies in ATCs/MTCs that reported rates of cranial CT scans. The overall pooled rate of CT scans across all studies was 38.5%

(95% CI: 27.7%—50.6%), with a high level of heterogeneity (I^2^ = 99.1%) (Fig. 4).

**Fig. 5:**
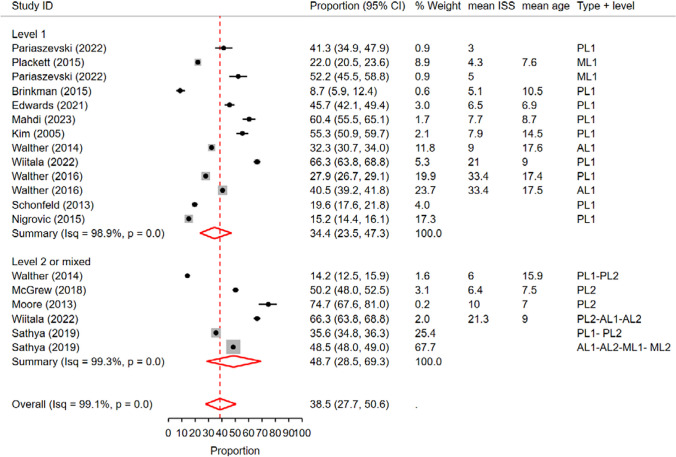
Forest plot of the random effects meta-analysis of the proportion of pediatric blunt trauma cases receiving cranial CT scans, stratified by trauma center level. The analysis includes studies conducted in level 1 trauma centers and level 2 trauma centers, as well as studies reporting rates in a combination of both levels. The studies within each subgroup are arranged based on the mean Injury Severity Score. AL1&2: Adult Level 1 & 2 trauma centers. CI: Confidence Interval. ISS: Injury Severity Score. ML1&2: Mixed Level 1 and 2 trauma centers. PL1&2: Pediatric Level 1 & 2 trauma centers

Metaregression analysis examining the effect of trauma center type showed a significant impact on the observed rates (p < 0.01, delta BIC = 1125.3). In subgroup analysis, the pooled rate of cranial CT scans in PTCs was 36.9% (95% CI: 25.3%—50.3%), while in ATCs/MTCs, it was 42.9% (95% CI: 31.4%—55.3%) (Fig. 4). Similarly, metaregression analysis considering trauma center level revealed a significant effect (p < 0.001, delta BIC = 103.6), with level 2 or mixed trauma centers demonstrating a higher pooled rate of 48.7% (95% CI: 28.5%—69.3%) compared to level 1 trauma centers, which had a pooled rate of 34.4% (95% CI: 23.5%—47.3%) (Fig. 5).

Furthermore, metaregression analysis of mean ISS scores indicated a significant effect on the reported rates (p < 0.01, delta BIC = 176.2), with studies having higher mean ISS scores showing higher rates of cranial CT scans. Similarly, metaregression analysis of mean age demonstrated a significant effect (p < 0.01, delta BIC = 220.1), with studies having higher mean ages showing lower CT rates (Figs. 2.1 and 2.2 of Supplementary File). However, metaregression did not reveal a significant effect of publication year on the observed rates (p = 0.4).

In the bivariable metaregression considering trauma center type and level, both factors showed significant effects (trauma center type: p < 0.01, delta BIC = 1106.6; trauma center level: p < 0.01, delta BIC = 85). The effect of trauma center type remained significant after controlling for mean age (p < 0.01, delta BIC = 129.1) and mean ISS (p < 0.01, delta BIC = 164). The effect of trauma center level remained marginally significant after controlling for ISS score (p = 0.03, delta BIC = 2.3). However, this effect did not remain significant after controlling for mean age (p = 0.3). It is important to note that the bivariate metaregression controlling for mean ISS included 15 studies, while the bivariate metaregression for mean age included only 13 studies.

**Fig. 6:**
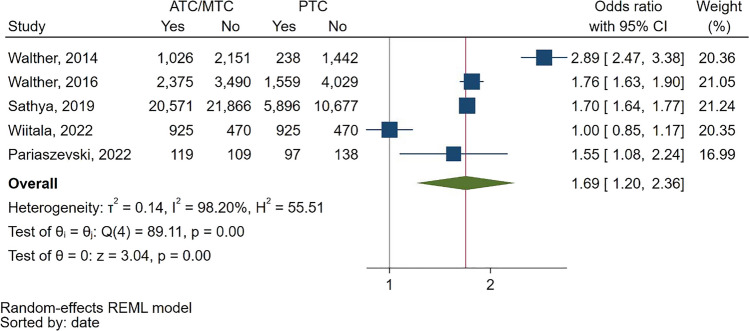
Forest plot of the random effects odds ratio meta-analysis of five studies that comparatively report the rates of cranial CT scans in pediatric blunt trauma patients in adult or mixed trauma centers compared to exclusively pediatric trauma centers. ATC: Adult Trauma Center. CI: Confidence Interval. MTC: Mixed Trauma Center. PTC: Pediatric Trauma Center

Consistent with the metaregression results in the proportion meta-analysis, the comparative OR meta-analysis of studies reporting rates in pediatric and other trauma centers showed that ATCs/MTCs had a significantly higher cranial CT rate compared to PTCs, with a pooled OR of 1.69 (95% CI: 1.2—2.36) (Fig. 6).

#### Chest CT scans

**Fig. 7:**
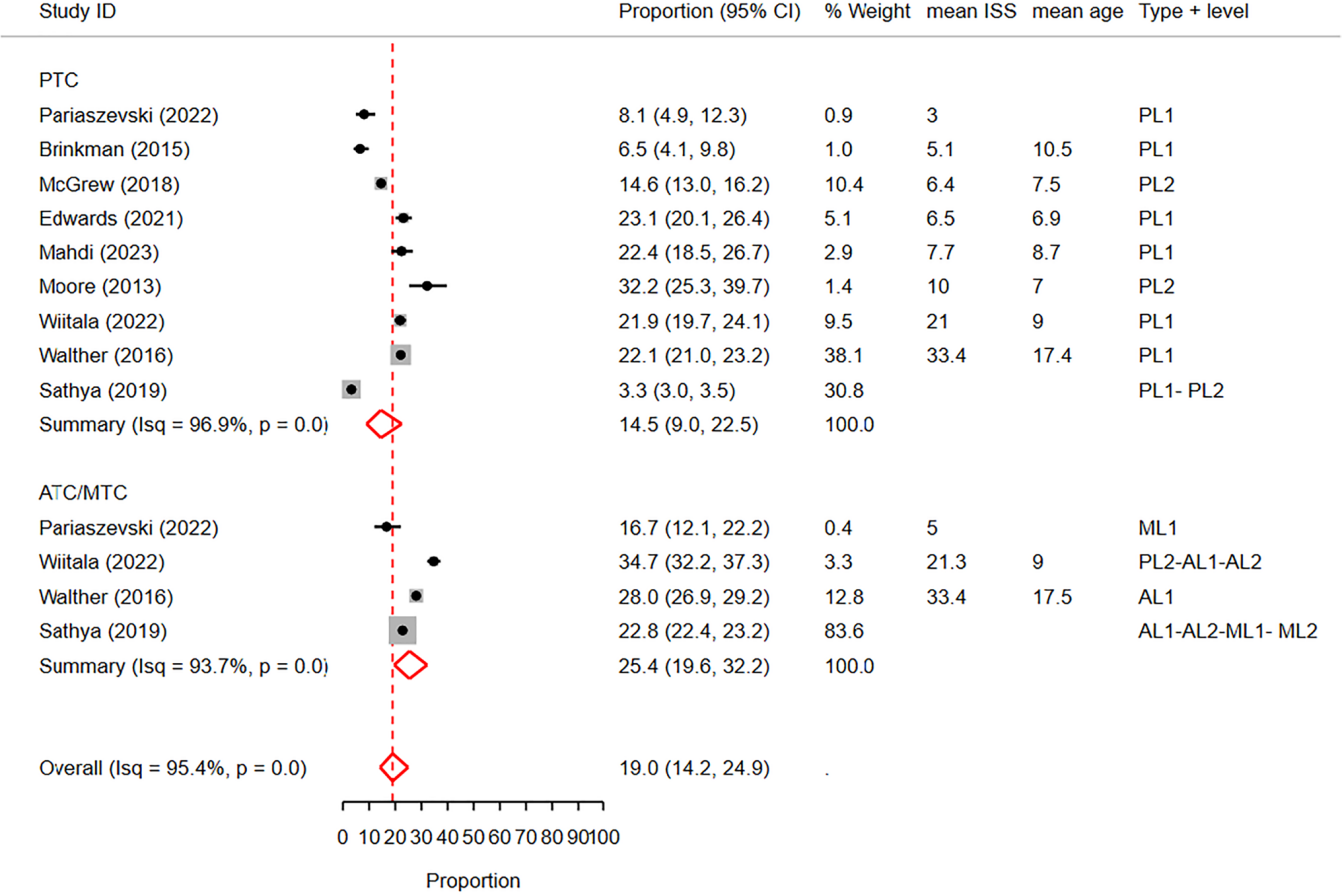
Forest plot of the random effects meta-analysis of the proportion of pediatric blunt trauma cases receiving chest CT scans in pediatric trauma centers and adult/mixed trauma centers. The studies within each subgroup are arranged based on the mean Injury Severity Score. AL1&2: Adult Level 1 & 2 trauma centers. ATC: Adult trauma center. CI: Confidence Interval. ISS: Injury Severity Score. ML1&2: Mixed Level 1 and 2 trauma centers. MTC: Mixed Trauma Center. PL1&2: Pediatric Level 1 & 2 trauma centers. PTC: Pediatric Trauma Center

A total of 15 studies were included in our analysis: 10 studies conducted in PTCs and 5 studies in ATCs/MTCs. Through proportion meta-analysis, we found that the pooled rate of chest CT scans was 19% (95% CI: 14.2%—24.9%) with significant heterogeneity (I^2^ = 95.4%) (Fig. 7).

When exploring the effect of trauma center type through metaregression, we observed a significant impact on the reported rates (p < 0.01, delta BIC = 3150.2). In subgroup analysis, the pooled rate of chest CT scans in PTCs was 14.5% (95% CI: 9%—22.5%), while in ATCs/MTCs, the pooled rate was 25.4% (95% CI: 19.6%—32.2%) (Fig. 7).

**Fig. 8:**
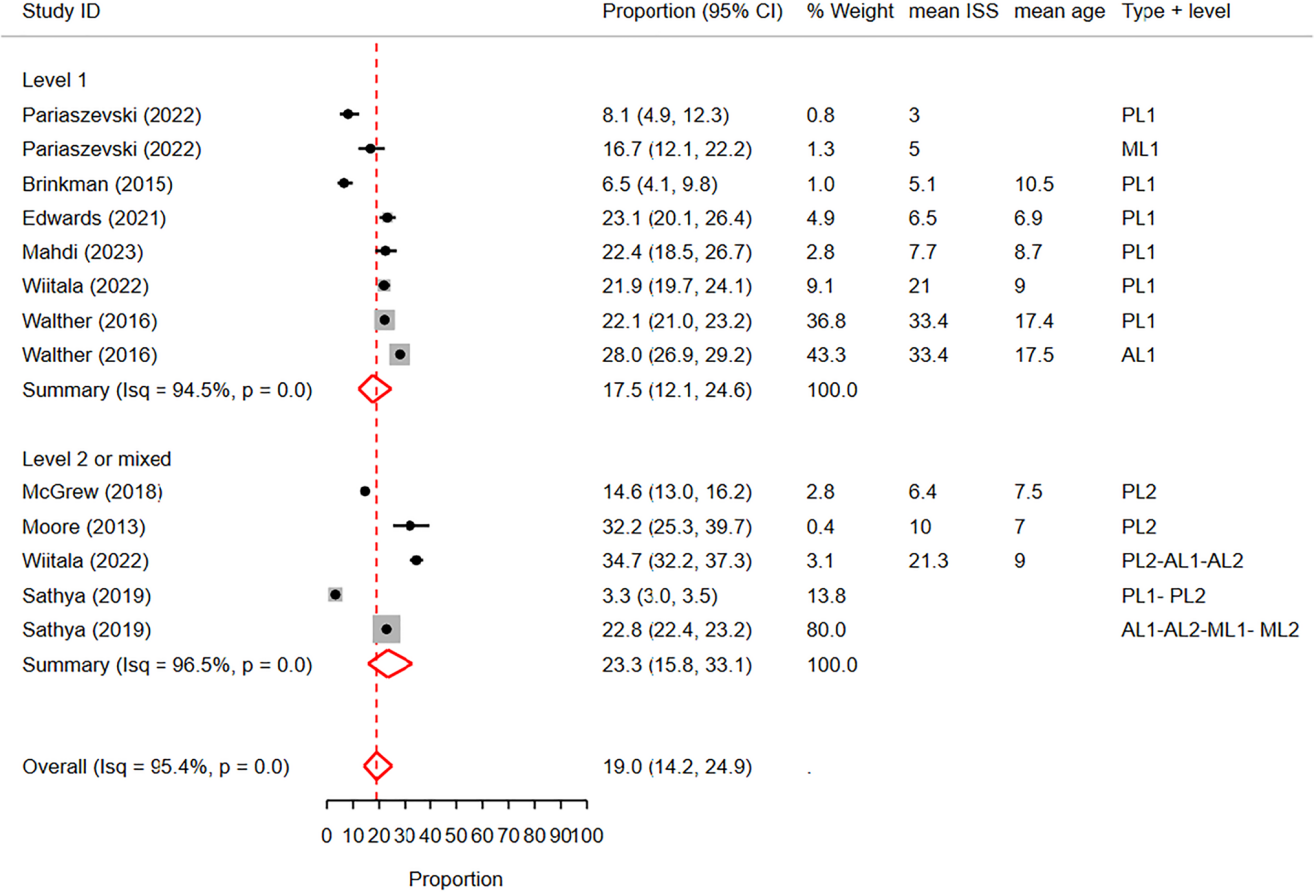
Forest plot of the random effects meta-analysis of the proportion of pediatric blunt trauma cases receiving chest CT scans, stratified by trauma center level. The analysis includes studies conducted in level 1 trauma centers and level 2 trauma centers, as well as studies reporting rates in a combination of both levels. The studies within each subgroup are arranged based on the mean Injury Severity Score. AL1&2: Adult Level 1 & 2 trauma centers. CI: Confidence Interval. ISS: Injury Severity Score. ML1&2: Mixed Level 1 and 2 trauma centers. PL1&2: Pediatric Level 1 & 2 trauma centers

Similarly, metaregression and subgroup analysis based on trauma center level revealed a significantly lower chest CT rate in level 1 trauma centers (pooled rate: 17.5%, 95% CI: 12.1%—24.6%) compared to level 2 or mixed centers (pooled rate: 23.3%, 95% CI: 15.8%—33.1%) (p < 0.01, delta BIC = 54.1) (Fig. 8).

Furthermore, metaregression analysis of the mean ISS demonstrated a significant effect on the observed chest CT rates, indicating a trend toward higher rates in studies with higher mean ISS scores (p < 0.02, delta BIC = 3.5) (Fig. 3 of Supplementary File). However, univariable metaregression did not show any significant correlation between mean age or publication year and chest CT rates (p = 0.3 and p = 0.8, respectively).

In bivariable metaregression with trauma center types and levels as covariates, both trauma center type (p < 0.01, delta BIC = 3156.1) and trauma center level (p < 0.01, delta BIC = 60) showed significant effects. It is important to note that the number of studies included in this bivariate analysis was relatively small (N = 13).

**Fig. 9:**
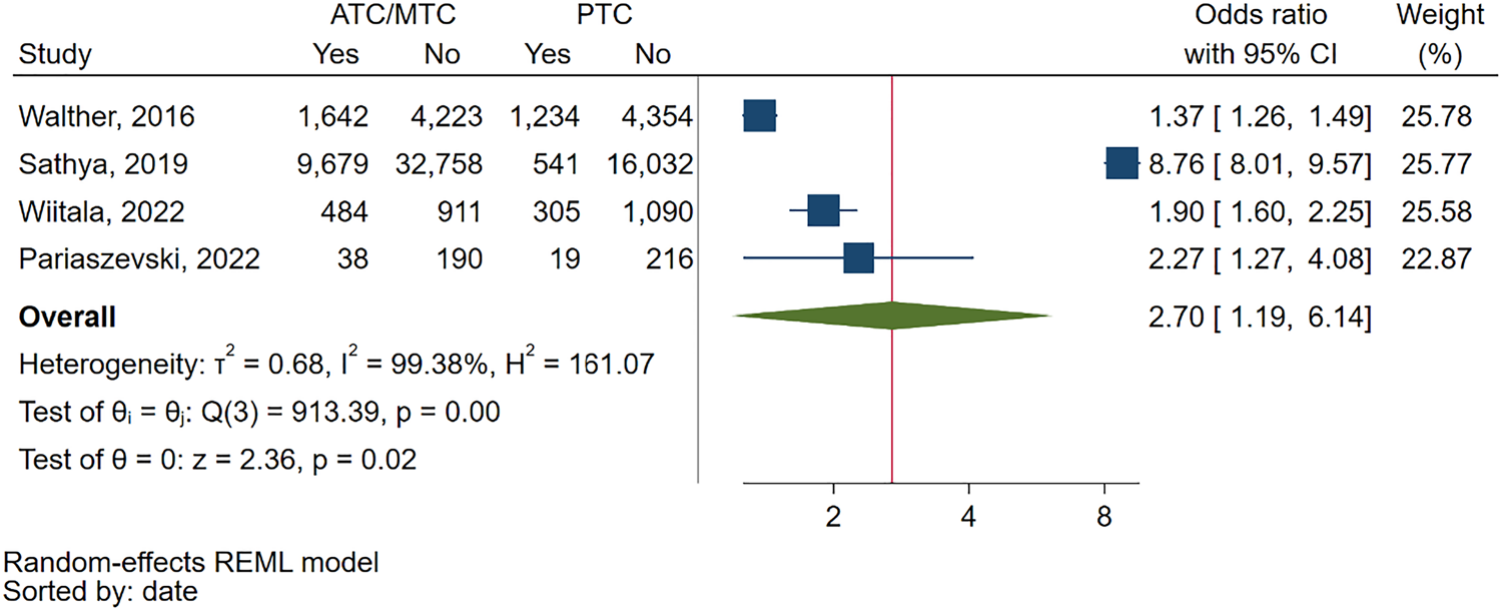
Forest plot of the random effects odds ratio meta-analysis of four studies that comparatively report the rates of chest CT scans in pediatric blunt trauma patients in adult or mixed trauma centers compared to exclusively pediatric trauma centers. ATC: Adult Trauma Center. CI: Confidence Interval. MTC: Mixed Trauma Center. PTC: Pediatric Trauma Center

Lastly, in the OR meta-analysis of 4 comparative studies reporting chest CT rates, we found that ATCs/MTCs had a significantly higher chest CT rate compared to pediatric centers, with a pooled OR of 2.7 (95% CI: 1.19—6.14) (Fig. 9).

#### Cervical spine CT scans

**Fig. 10:**
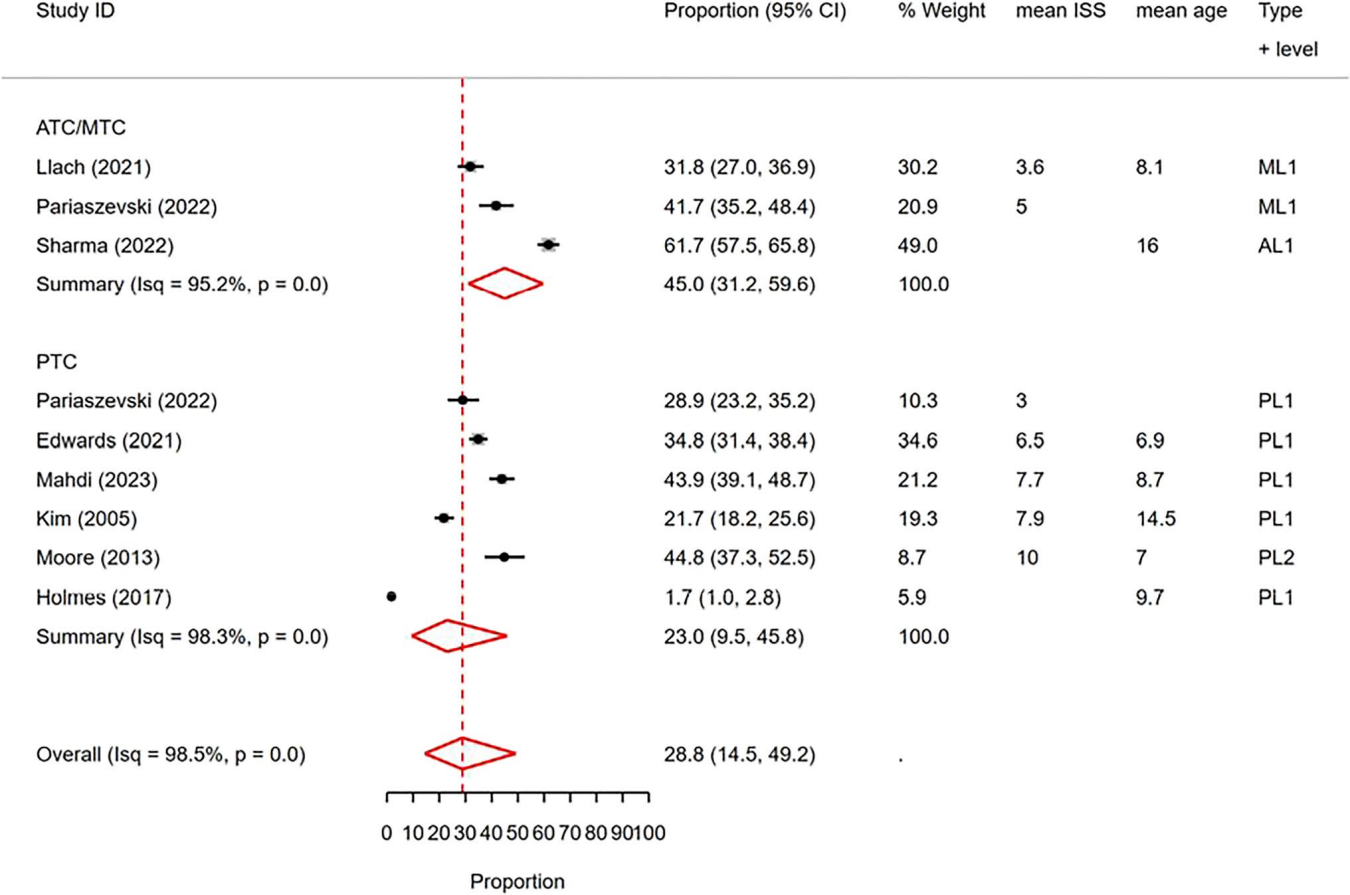
Forest plot of the random effects meta-analysis of the proportion of pediatric blunt trauma cases receiving cervical CT scans in pediatric trauma centers and adult/mixed trauma centers. The studies within each subgroup are arranged based on the mean Injury Severity Score. AL1&2: Adult Level 1 & 2 trauma centers. ATC: Adult trauma center. CI: Confidence Interval. ISS: Injury Severity Score. ML1&2: Mixed Level 1 and 2 trauma centers. MTC: Mixed Trauma Center. PL1&2: Pediatric Level 1 & 2 trauma centers. PTC: Pediatric Trauma Center

We included a total of 12 studies that reported rates of cervical CT scans in our analysis, comprising 5 studies conducted in PTCs and 7 studies in ATCs/MTCs. The proportion meta-analysis of these reported rates revealed a pooled rate of 28.8% (95% CI: 14.5%—49.2%) with a high level of heterogeneity (I^2^ = 98.5%) (Fig. 10).

When conducting metaregression analysis with trauma center type as a covariate, we found a significant effect of trauma center type on the observed rates (p < 0.01, delta BIC = 7). In the subgroup meta-analysis, the pooled rate of cervical spine CT scans in PTCs was estimated to be 23% (95% CI: 9.5%—45.8%), while in ATCs/MTCs, the pooled rate was 45% (95% CI: 31.2%—59.6%) (Fig. 10). However, due to the limited availability of studies reporting cervical CT rates in level 2 or mixed-level trauma centers, it was not possible to perform metaregression or subgroup meta-analysis based on trauma center level.

Furthermore, in the univariable metaregression analysis, we considered mean ISS, mean age, and publication year as covariates. Among these, only mean ISS showed a significant effect (p < 0.05, delta BIC = 2.7), suggesting a trend towards higher cervical spine CT rates in studies with higher mean ISS upon visual inspection (Fig. 4 of Supplementary File). Unfortunately, the limited number of studies prevented the conduction of a multivariable meta-regression analysis and OR meta-analysis for this outcome.

#### Receiving at least one CT scan

**Fig. 11:**
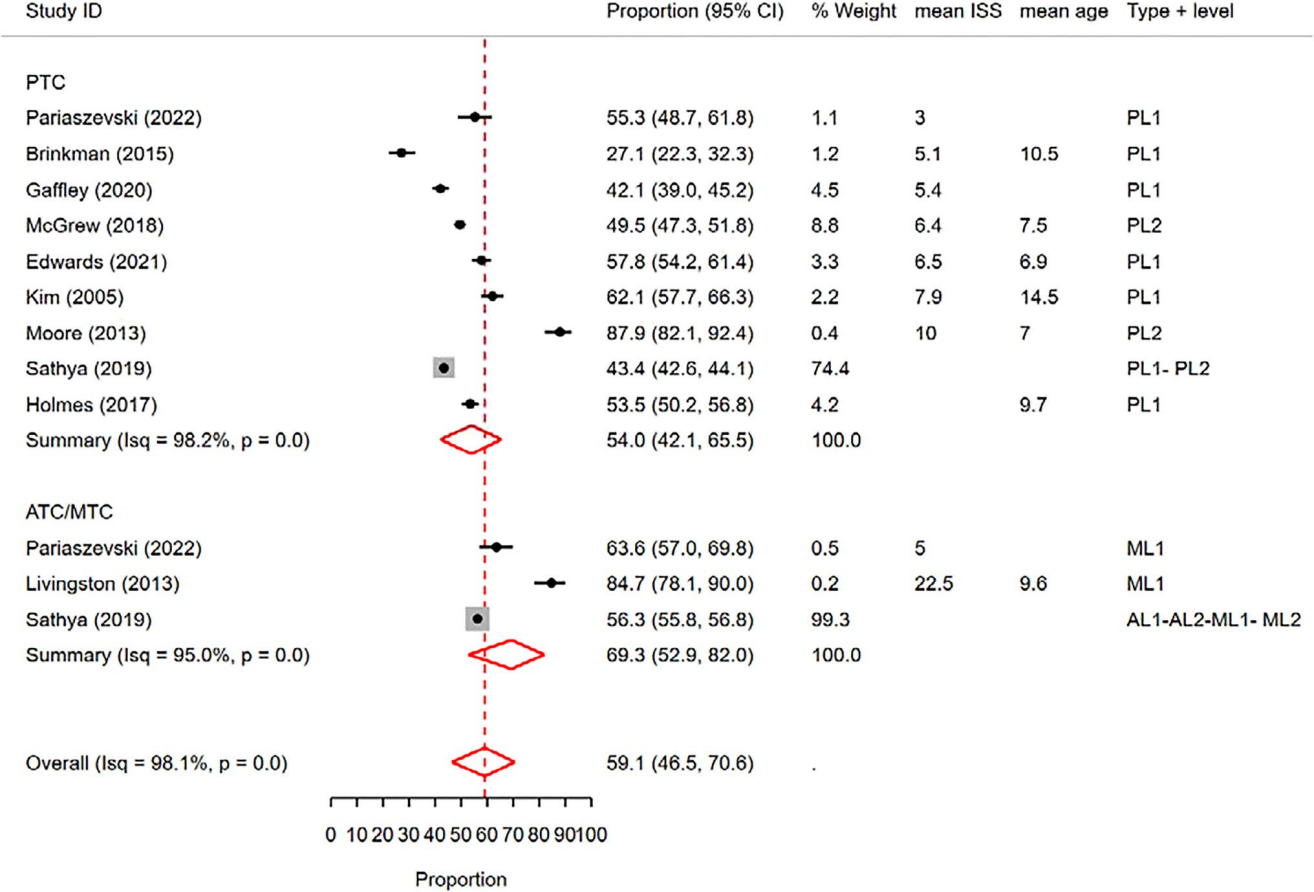
Forest plot of the random effects meta-analysis of the proportion of pediatric blunt trauma cases receiving at least one CT scan (of any type) in pediatric trauma centers and adult/mixed trauma centers. The studies within each subgroup are arranged based on the mean Injury Severity Score. AL1&2: Adult Level 1 & 2 trauma centers. ATC: Adult trauma center. CI: Confidence Interval. ISS: Injury Severity Score. ML1&2: Mixed Level 1 and 2 trauma centers. MTC: Mixed Trauma Center. PL1&2: Pediatric Level 1 & 2 trauma centers. PTC: Pediatric Trauma Center

Among the studies included in our analysis, 10 studies reported rates of receiving at least one CT scan (of any type). The proportion meta-analysis of these reported rates yielded a pooled rate of 59.1% (95% CI: 46.5%—70.6%) (Fig. 11). Similar to other reported rates, there was considerable heterogeneity among the included studies (I^2^ = 98.5%).

Upon conducting a meta-regression analysis, we found a significant effect of trauma center type on the rates (p < 0.05, delta BIC = 799.9). Subsequently, the subgroup meta-analysis demonstrated a pooled rate of 54% (95% CI: 42.1%—65.5%) for PTCs, while ATCs/MTCs had a pooled rate of 69.3% (95% CI: 52.9%—82%) (Fig. 11). However, the meta-regression analysis for the effect of trauma center level did not reveal any significant effect (p = 0.5).

Furthermore, we performed univariable meta-regression analyses to examine the effects of mean age, mean ISS, and publication year. Among these variables, only ISS showed a significant association with the heterogeneity of the observed rates (p < 0.01, delta BIC = 6.3). Visual inspection of the forest plots, sorted by mean ISS, indicated a trend towards higher rates in studies with higher ISS scores (Fig. 5 of Supplementary File). However, due to the limited number of studies reporting ISS scores along with the rates of CT use, a multivariable meta-regression analysis was not conducted for this outcome.

### Publication bias

The Doi plots representing the proportion meta-analyses for each outcome are presented in Figs. 6.1–6.7 of Supplementary File. Additionally, funnel plots illustrating the OR meta-analysis can be found in Figs. 7.1–7.3 of Supplementary File. When assessing publication bias using the LFK index for the proportion outcomes, minor asymmetry was observed in the cervical CT rates and the rates of receiving at least one CT scan (Figs. 6.4 and 6.5 of Supplementary File). However, significant asymmetry was noted in the Doi plot for the rates of abdominopelvic CT scans (Supplementary Fig. 6.1). Similarly, the funnel plot for the OR meta-analysis of abdominopelvic CT rates showed asymmetry. To address this, we conducted a nonparametric trim-and-fill analysis specifically for OR meta-analysis of abdominopelvic CT rates (Fig. 7.1 of Supplementary File). However, the inclusion of additional studies did not alter the significance level of the observed outcome, and the pooled OR remained at 2.1 (95% CI: 1.6—2.9) following imputation.

## Discussion

This systematic review and meta-analysis aimed to investigate disparities in CT utilization between PTCs and non-PTCs. The study revealed notable discrepancies in the propensity for pediatric blunt trauma patients to undergo various types of CT scans, such as abdominopelvic, cranial, chest, and cervical spine scans, contingent upon the trauma center's classification. Notably, PTCs demonstrated decreased rates of CT scans in comparison to ATCs/MTCs. These findings underscore the potential influence of the trauma center type on the utilization of CT scans in the management of pediatric trauma patients.

The decision to perform CT scans in pediatric patients is based on several factors, including the mechanism of injury, clinical presentation, physical examination findings, and the specific guidelines followed by the trauma center [47–49]. Pediatric patients, particularly infants and young children, are more sensitive to radiation exposure compared to adults. Their increased radiosensitivity stems from their developing bodies, higher metabolic rates, and longer life expectancies, which provide more time for potential radiation-related effects to manifest [6, 25, 50]. Therefore, guidelines for pediatric CT imaging generally aim to minimize unnecessary radiation exposure [51].

Pediatric trauma centers often follow guidelines specific to the pediatric population, such as those developed by the American College of Surgeons (ACS) Pediatric Trauma Quality Improvement Program (TQIP) [52, 53]. These guidelines consider age-specific considerations, injury patterns common in children, and the potential risks associated with radiation exposure. They typically emphasize clinical assessment and observation before considering CT scans. In a study involving 3,832 pediatric patients with minor head injuries, the utilization of the PECARN rule alone led to a 29% reduction in the rate of head CT scans, all without overlooking clinically significant head traumas [54]. Another multicentric study on pediatric head trauma indicated that the Pediatric Emergency Care Applied Research Network (PECARN) rule had potential to prevent 396 out of 589 unnecessary head CT scans [55]. Notably, Wu et al. reported a significant 50% reduction in the number of CT scans ordered for pediatric trauma patients through a 3-month evidence-based imaging education program designed for emergency and surgery practitioners [56].

Variations in clinical approaches and the unique preferences of individual physicians within each trauma center can also influence the decision-making process regarding the application of CT imaging. The degree of underestimating radiation risks may also diverge among physicians in ATCs/ MTCs when compared to those in PTCs. While no study has directly compared this aspect across different trauma centers, it is worth noting that educational interventions regarding radiation risks have demonstrated the potential to reduce the frequency of CT scan orders. A study involving 21 residents revealed that after attending lectures on radiation exposure associated with CT scans, 33% of participants opted for alternative imaging modalities [57].

In our analysis, a significant trend emerged, revealing elevated rates of abdominopelvic and cranial CT scans utilization in the younger age groups among pediatric patients with blunt trauma. The smaller body sizes and distinctive anatomical features of children render them more susceptible to the impacts of blunt trauma [58]. Consequently, this vulnerability may drive physicians to opt for CT scans as a means to thoroughly investigate potential injuries.

Moreover, caregivers' apprehensions regarding potential injuries can influence the decision of physicians to order CT scans. A study focusing on pediatric emergency fellowship program leaders revealed that 73% of them discussed radiation risks in the context of infant patients. According to the respondents, these discussions were often initiated in response to parents requesting unnecessary CT scans or expressing concerns about radiation risks [59]. The significance of addressing radiation risks associated with CT scans was underscored in a study involving 971 children with minor traumatic brain injuries. This study demonstrated that educating parents led to a reduction in the number of CT scans ordered in emergency departments. Additionally, patients whose parents received education underwent significantly fewer imaging tests within a week of hospitalization [24].

Among the studies encompassed in our analysis, three of them conducted a comparison of the mortality rates between children treated at PTCs and ATCs [1–3]. These studies revealed that despite the discrepancies in CT scan utilization among children treated at distinct trauma centers, there were no significant differences in mortality rates. Further studies could explore the potential impact of CT scan utilization on clinical outcomes for children treated at PTCs versus non-PTCs, aiming to elucidate the relationship between these factors.

Our primary objective was to compare the outcomes of interest in PTCs with non-PTCs. Due to the limited number of studies that exclusively included ATCs for most outcomes, we were unable to conduct specific comparisons between ATCs and MTCs, either with each other or with PTCs. Therefore, we combined the ATC and MTC data and compared them to a larger cohort of PTC data. However, this approach could introduce bias if there are significant differences between ATCs and MTCs in reporting the outcomes of interest. It is also important to acknowledge that incorporating studies from a range of countries and trauma center settings might introduce variations in data collection methods and clinical practices. This could impact the generalizability of our findings to specific regions or healthcare systems. Moreover, reliance on aggregated data from these studies limited our ability to conduct detailed analyses or adjust for individual patient characteristics that could impact the outcomes. Future studies should focus on understanding the underlying factors contributing to the observed differences in CT scan rates for pediatric blunt trauma patients between different trauma center types. It would be valuable to investigate the reasons behind the higher CT scan rates in trauma patients. Additionally, exploring the potential impact of other variables, such as clinical guidelines and physician experience, on CT scan rates would provide further insights into the decision-making process.

### Supplementary Information

Below is the link to the electronic supplementary material.

Supplementary file1 (DOCX 1583 KB)

## Data Availability

The datasets analyzed during the current study are available from the corresponding author on reasonable request.

## Declarations

### Leadership role

Ali Gholamrezanezhad serves as the Director of Clinical Research at the Department of Radiology in the Keck School of Medicine of USC.
