# Supplementary material for: Disparities in computed tomography utilization for pediatric blunt trauma: a systematic review and meta-analysis comparing pediatric and non-pediatric trauma centers
Source: Emerg Radiol. 2023 Sep 23;30(6):743–64. doi: 10.1007/s10140-023-02172-3 (PMC10695891; doi:10.1007/s10140-023-02172-3)
Supplement: Supplementary file 1 — Supplementary file1 (DOCX 1583 KB) [file 10140_2023_2172_MOESM1_ESM.docx]

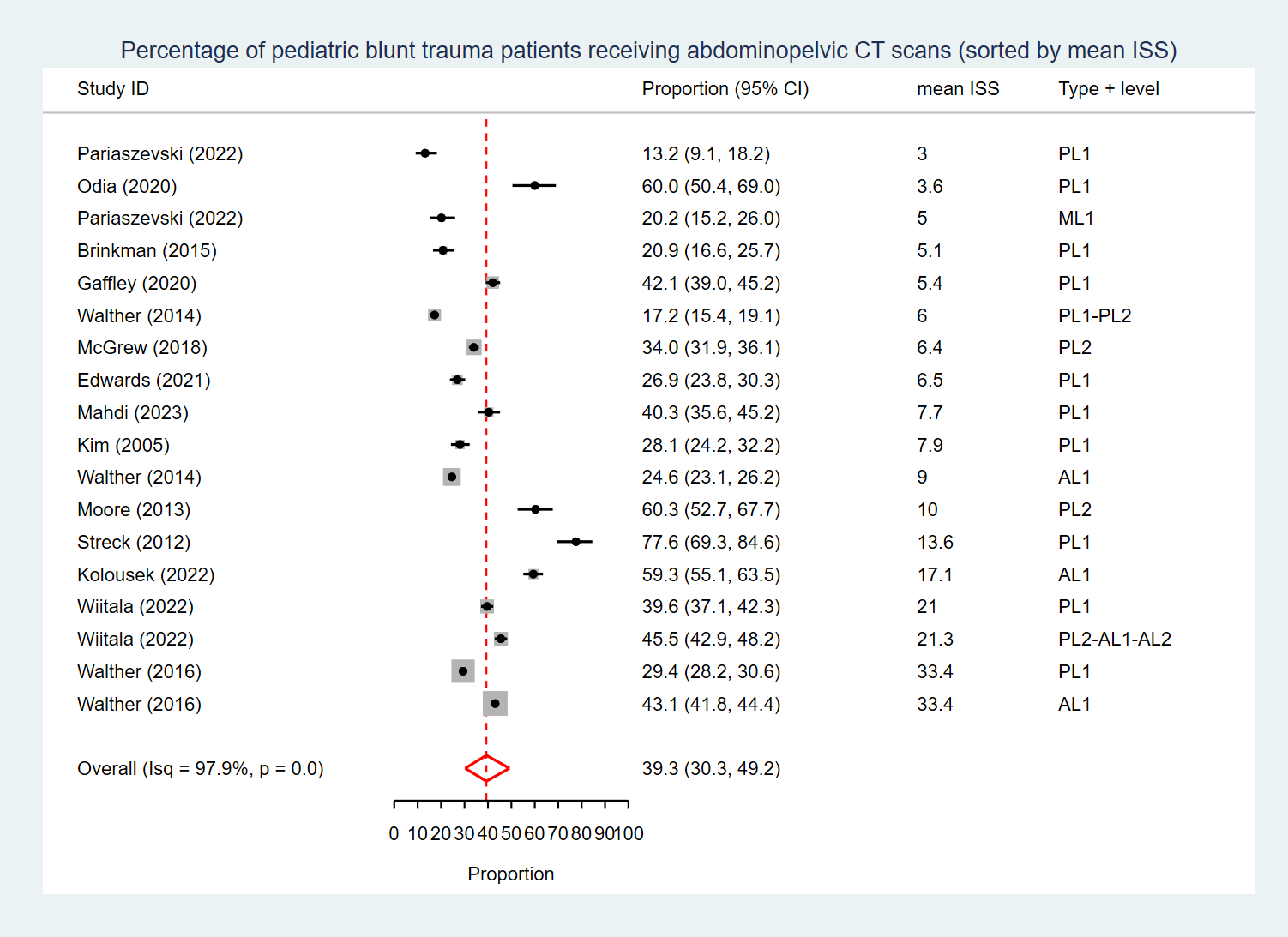


**Fig. 1.1** Forest plot of the random effects meta-analysis of the proportion of pediatric blunt trauma cases receiving abdominopelvic CT scans. The studies are sorted by mean injury severity score. AL1&2: Adult Level 1 & 2 trauma centers. CI: Confidence Interval. ISS: Injury Severity Score. ML1&2: Mixed Level 1 and 2 trauma centers. PL1&2: Pediatric Level 1 & 2 trauma centers


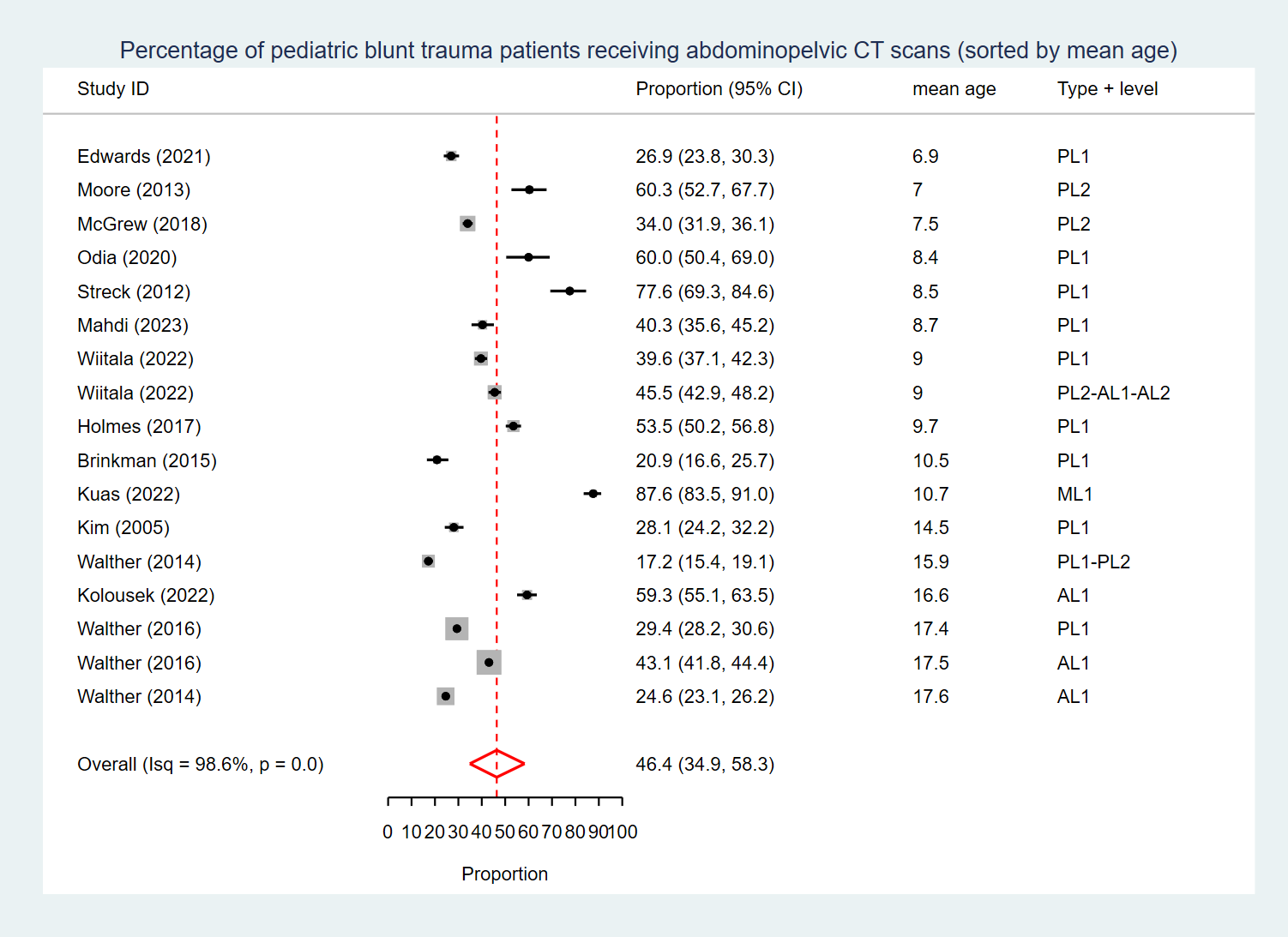


**Fig. 2.2** Forest plot of the random effects meta-analysis of the proportion of pediatric blunt trauma cases receiving abdominopelvic CT scans. The studies are sorted by mean age. AL1&2: Adult Level 1 & 2 trauma centers. CI: Confidence Interval. ML1&2: Mixed Level 1 and 2 trauma centers. PL1&2: Pediatric Level 1 & 2 trauma centers


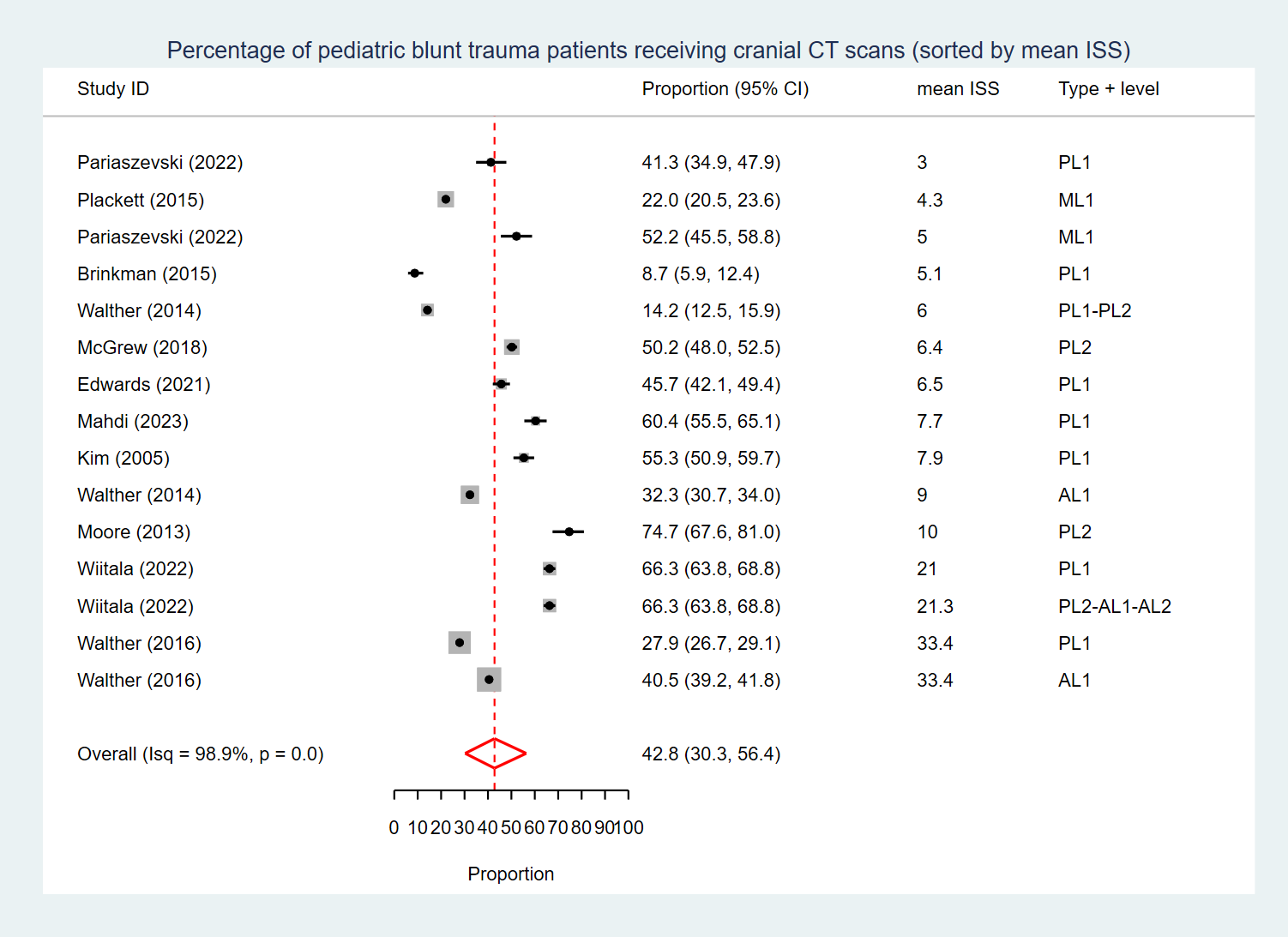


**Fig. 2.1** Forest plot of the random effects meta-analysis of the proportion of pediatric blunt trauma cases receiving cranial CT scans. The studies are sorted by mean injury severity score. AL1&2: Adult Level 1 & 2 trauma centers. CI: Confidence Interval. ISS: Injury Severity Score. ML1&2: Mixed Level 1 and 2 trauma centers. PL1&2: Pediatric Level 1 & 2 trauma centers


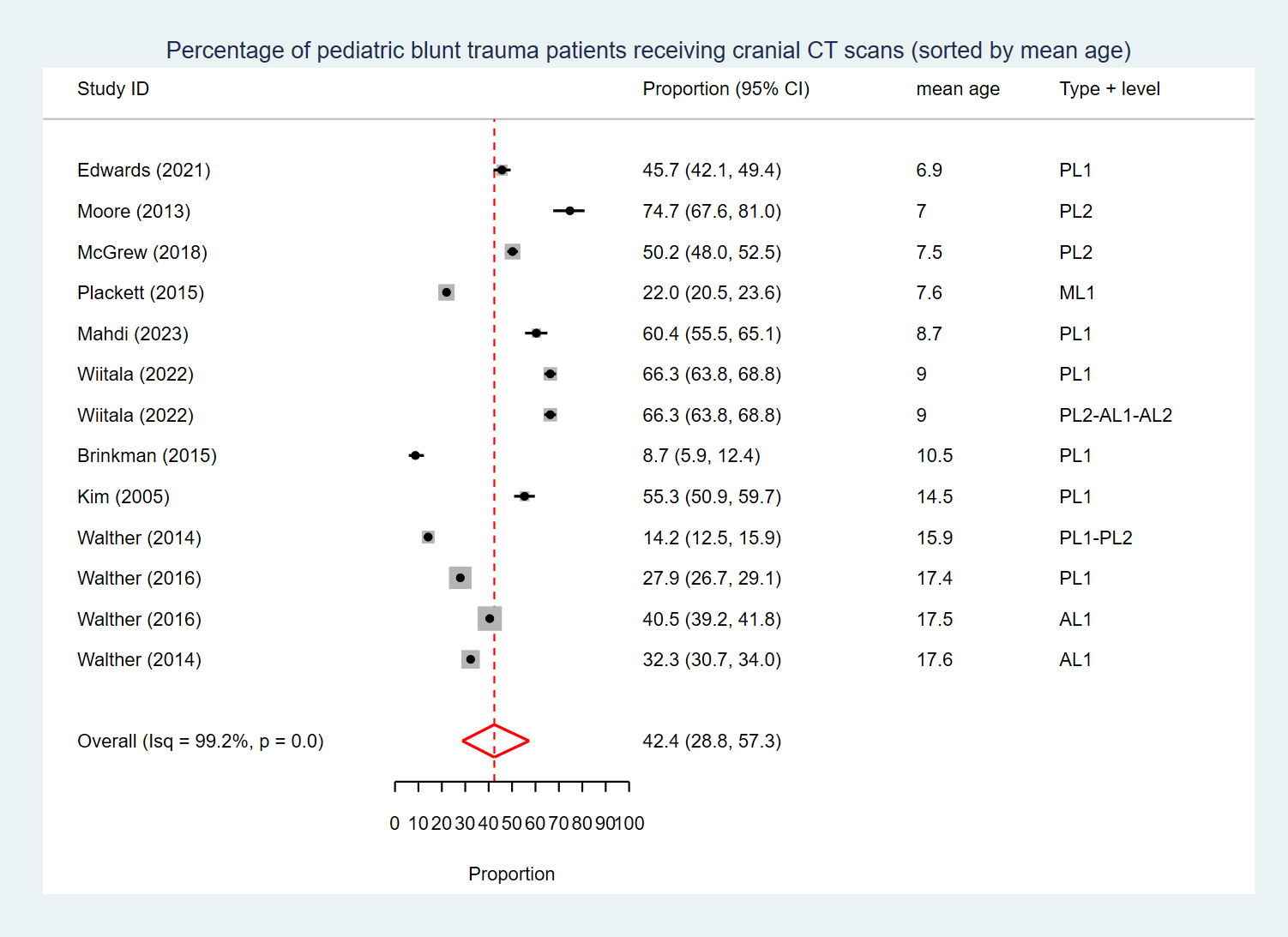


**Fig. 2.2** Forest plot of the random effects meta-analysis of the proportion of pediatric blunt trauma cases receiving cranial CT scans. The studies are sorted by mean age. AL1&2: Adult Level 1 & 2 trauma centers. ATC: Adult trauma center. CI: Confidence Interval. ML1&2: Mixed Level 1 and 2 trauma centers. MTC: Mixed Trauma Center. PL1&2: Pediatric Level 1 & 2 trauma centers. PTC: Pediatric Trauma Center


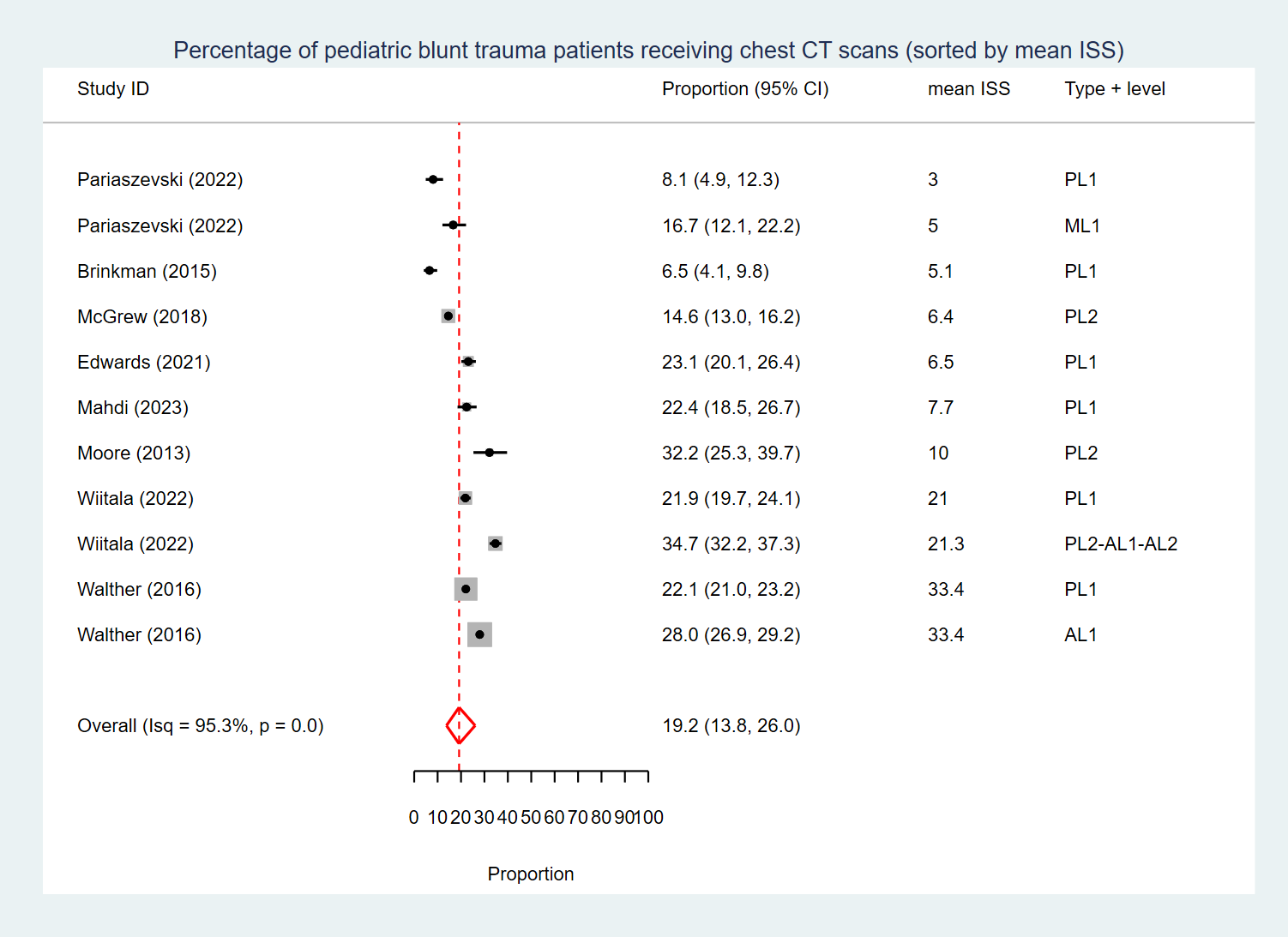


**Fig. 3** Forest plot of the random effects meta-analysis of the proportion of pediatric blunt trauma cases receiving chest CT scans. The studies are sorted by mean injury severity score. AL1&2: Adult Level 1 & 2 trauma centers. CI: Confidence Interval. ISS: Injury Severity Score. ML1&2: Mixed Level 1 and 2 trauma centers. PL1&2: Pediatric Level 1 & 2 trauma centers


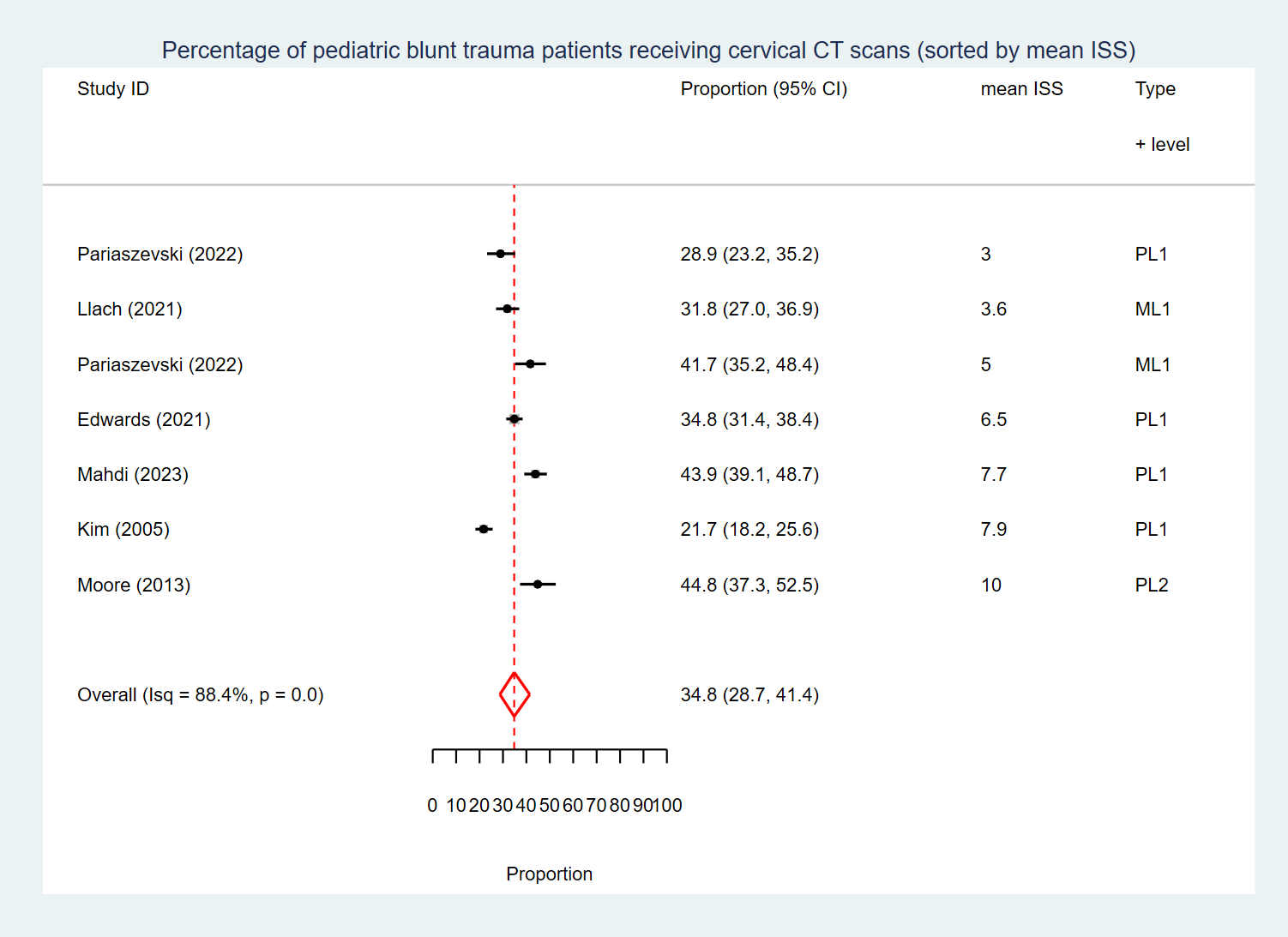


**Fig. 4** Forest plot of the random effects meta-analysis of the proportion of pediatric blunt trauma cases receiving cervical spine CT scans. The studies are sorted by mean injury severity score. AL1&2: Adult Level 1 & 2 trauma centers. CI: Confidence Interval. ISS: Injury Severity Score. ML1&2: Mixed Level 1 and 2 trauma centers. PL1&2: Pediatric Level 1 & 2 trauma centers


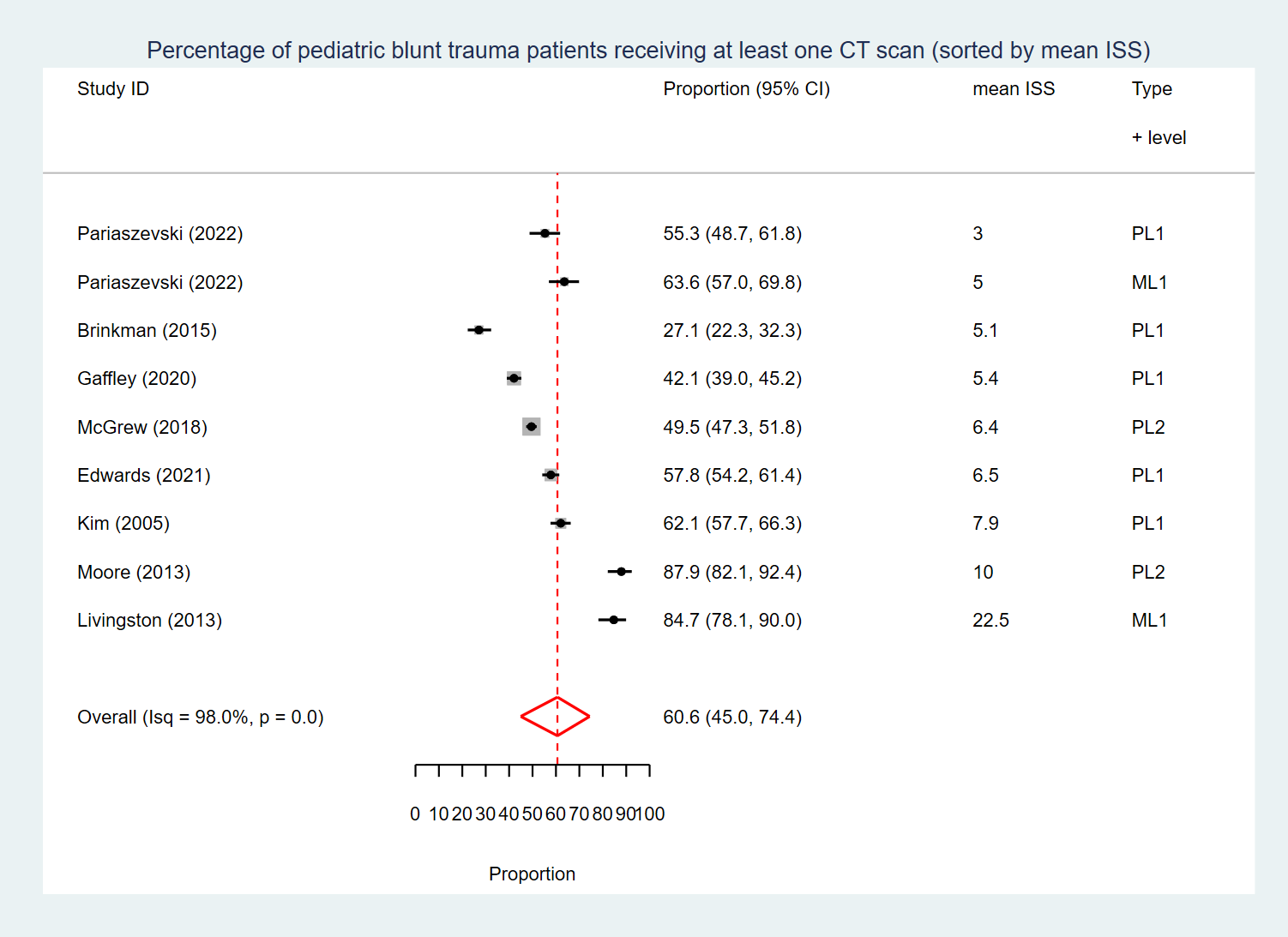


**Fig. 5** Forest plot of the random effects meta-analysis of the proportion of pediatric blunt trauma cases receiving at least one CT scan. The studies are sorted by mean injury severity score. AL1&2: Adult Level 1 & 2 trauma centers. CI: Confidence Interval. ISS: Injury Severity Score. ML1&2: Mixed Level 1 and 2 trauma centers. PL1&2: Pediatric Level 1 & 2 trauma centers


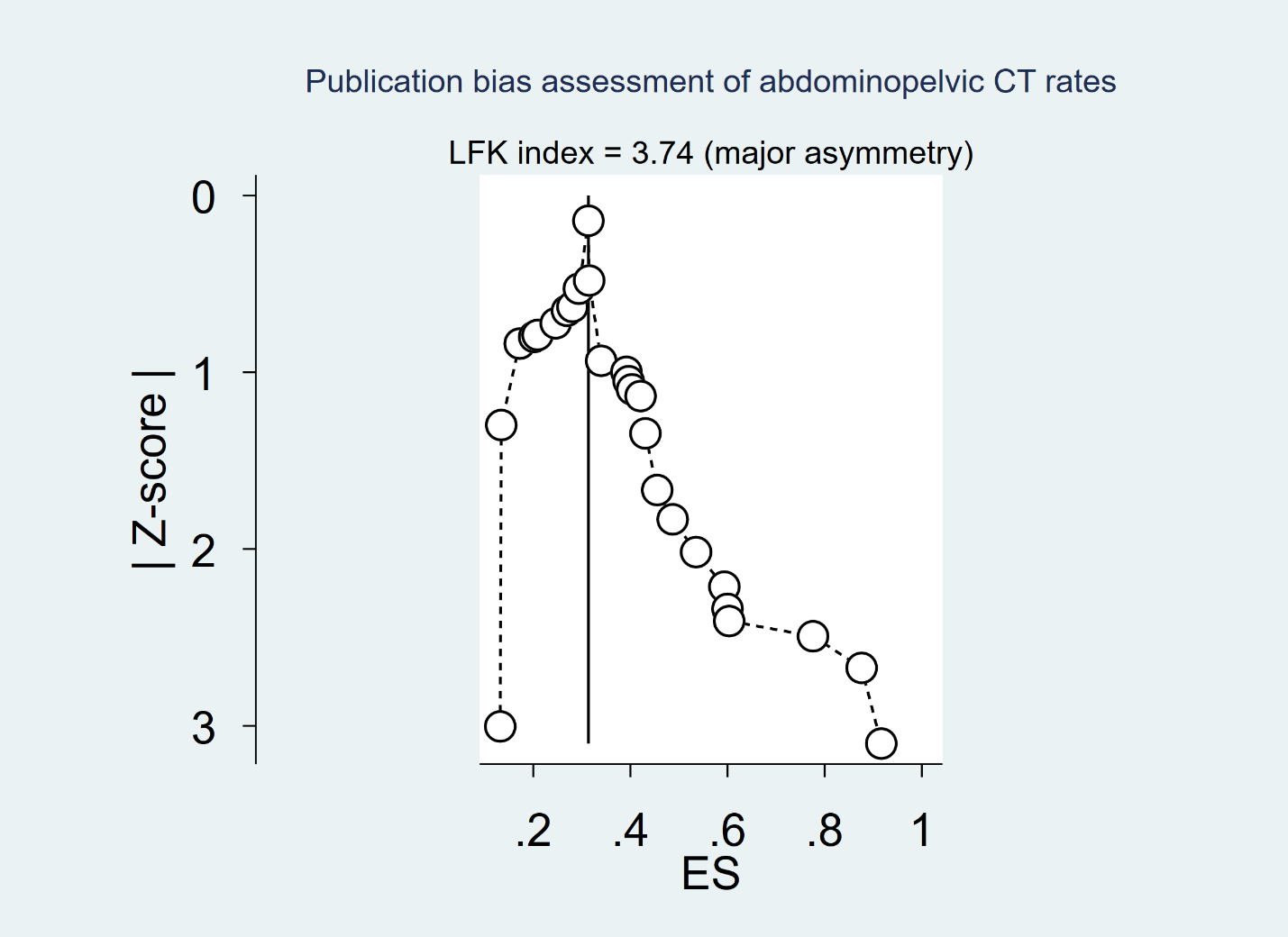


**Fig. 6.1** Doi plot for assessing publication bias of proportion meta-analysis of the rates of abdominopelvic CT scans. ES: Effect Size


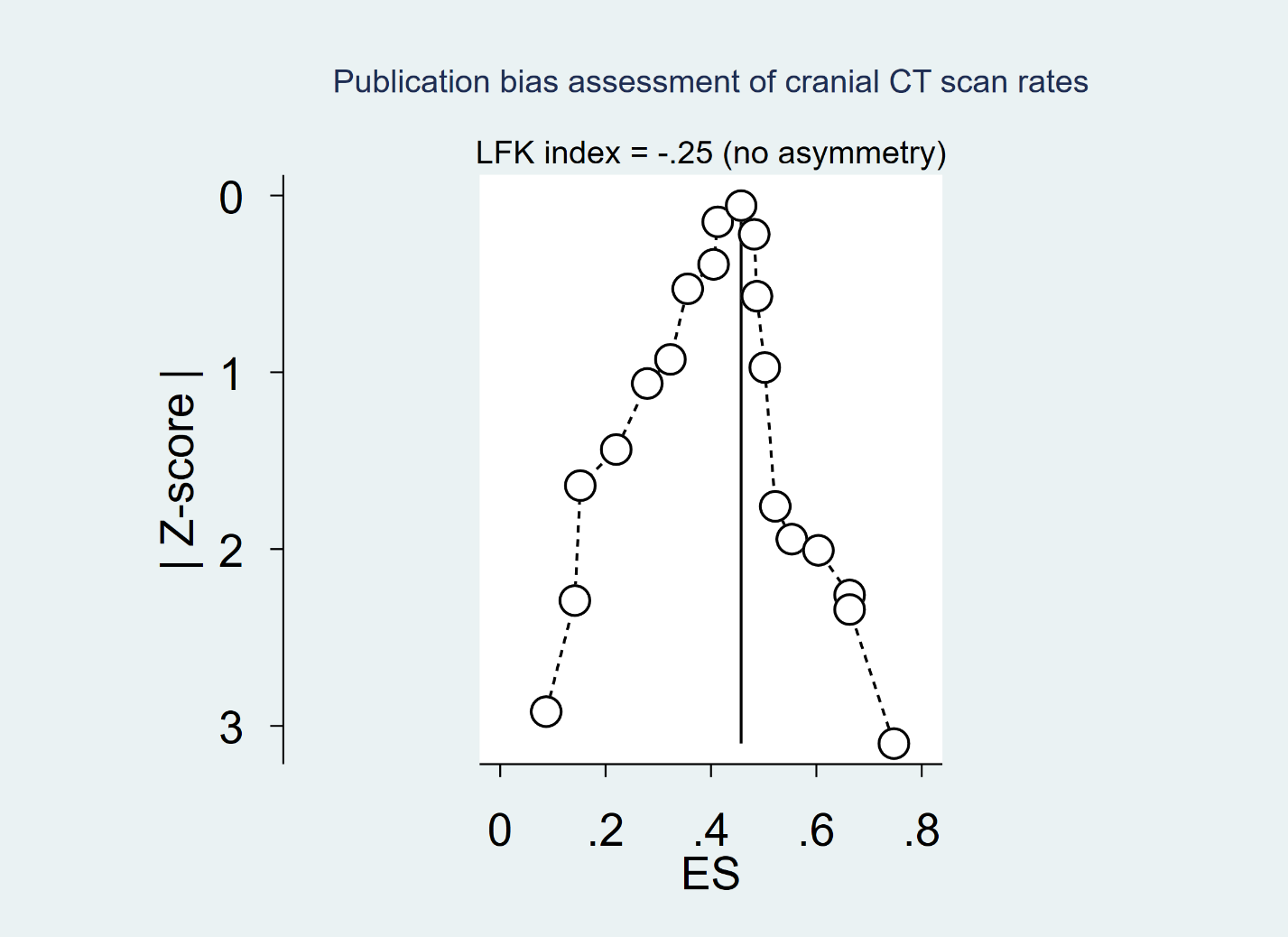


**Fig. 6.2** Doi plot for assessing publication bias of proportion meta-analysis of the rates of cranial CT scans. ES: Effect Size


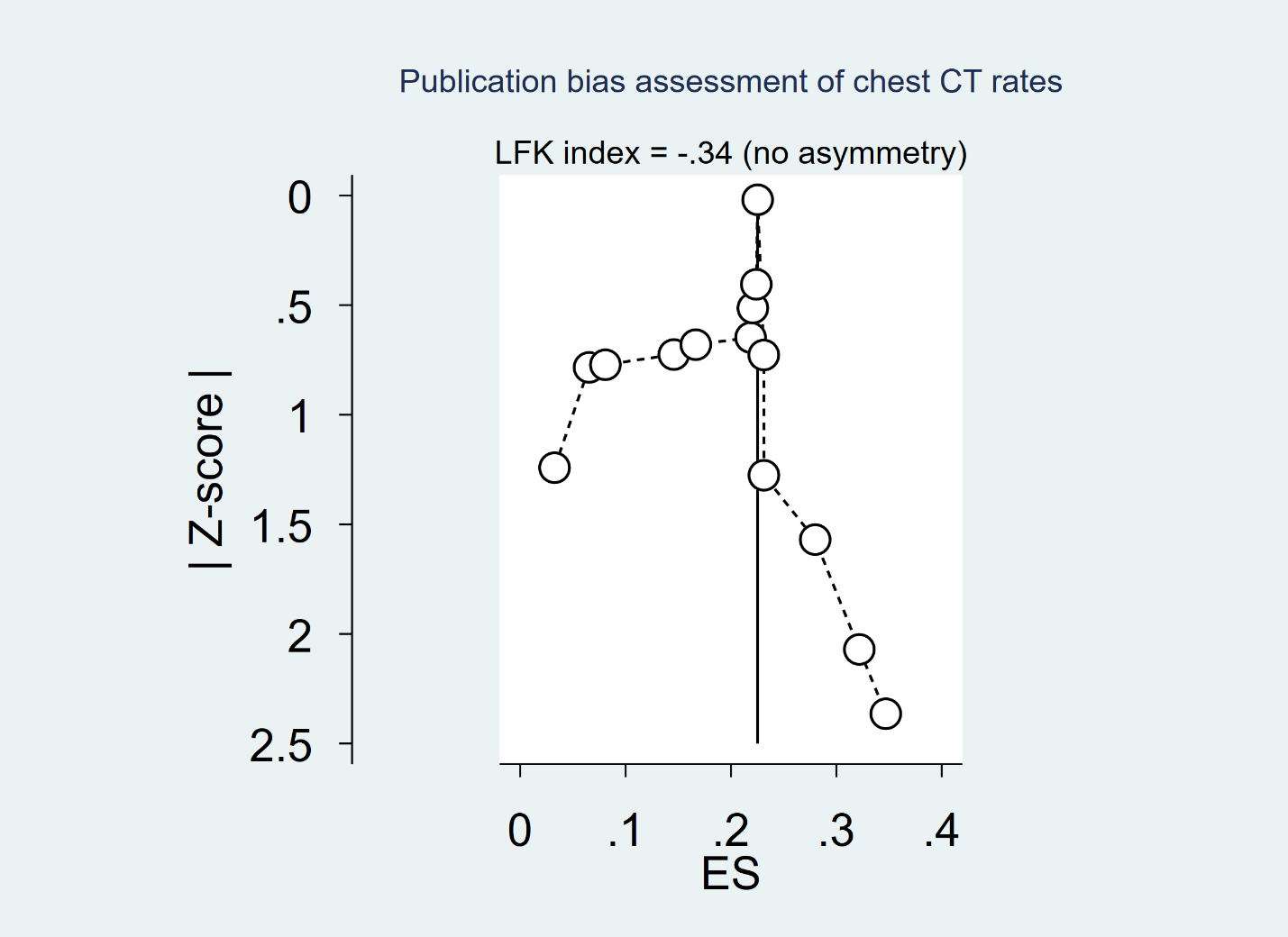


**Fig. 6.3** Doi plot for assessing publication bias of proportion meta-analysis of the rates of chest CT scans. ES: Effect Size


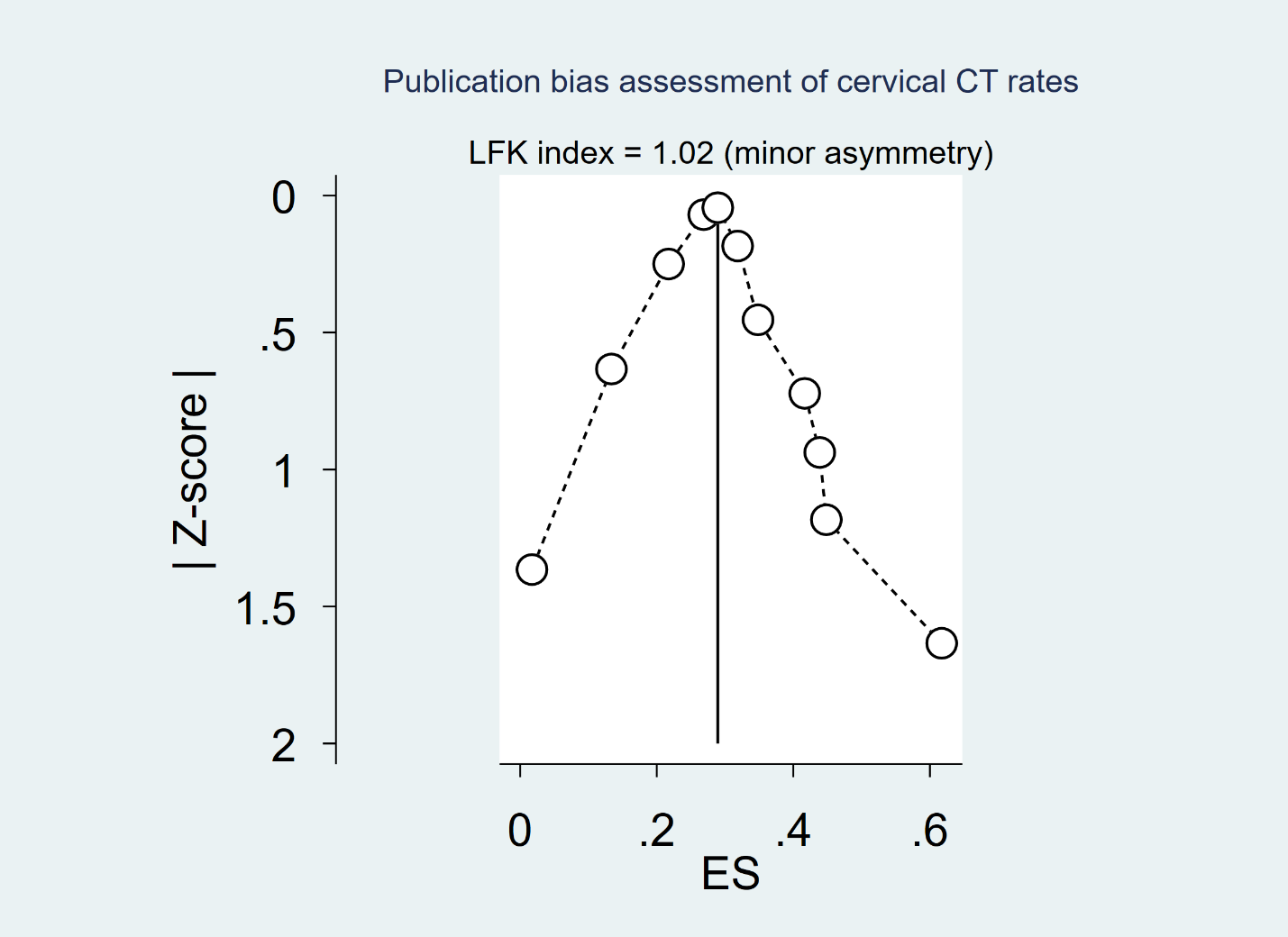


**Fig. 6.4** Doi plot for assessing publication bias of proportion meta-analysis of the rates of cervical CT scans. ES: Effect Size


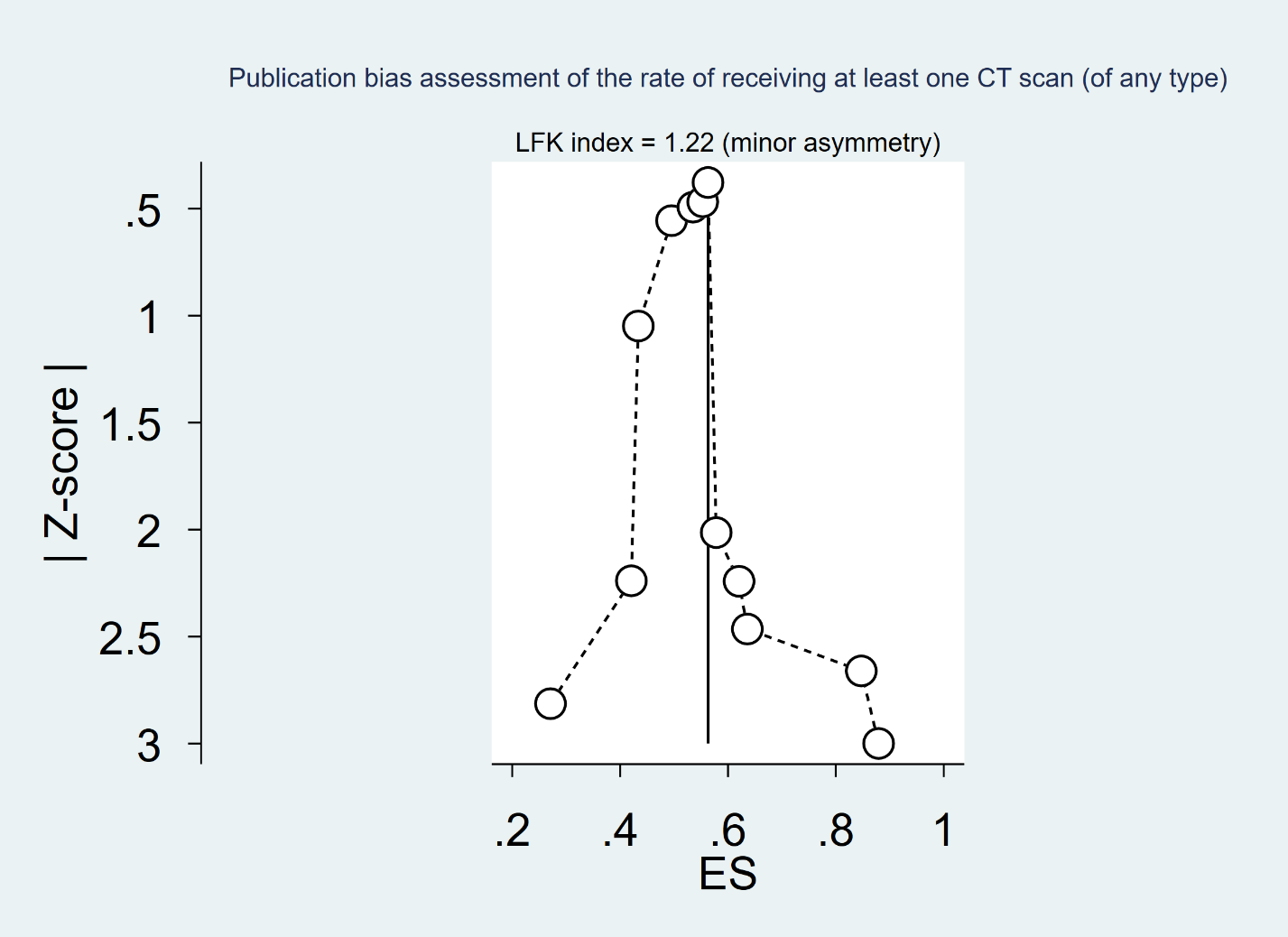


**Fig. 6.5** Doi plot for assessing publication bias of proportion meta-analysis of the rates of receiving at least one CT scan (of any type). ES: Effect Size


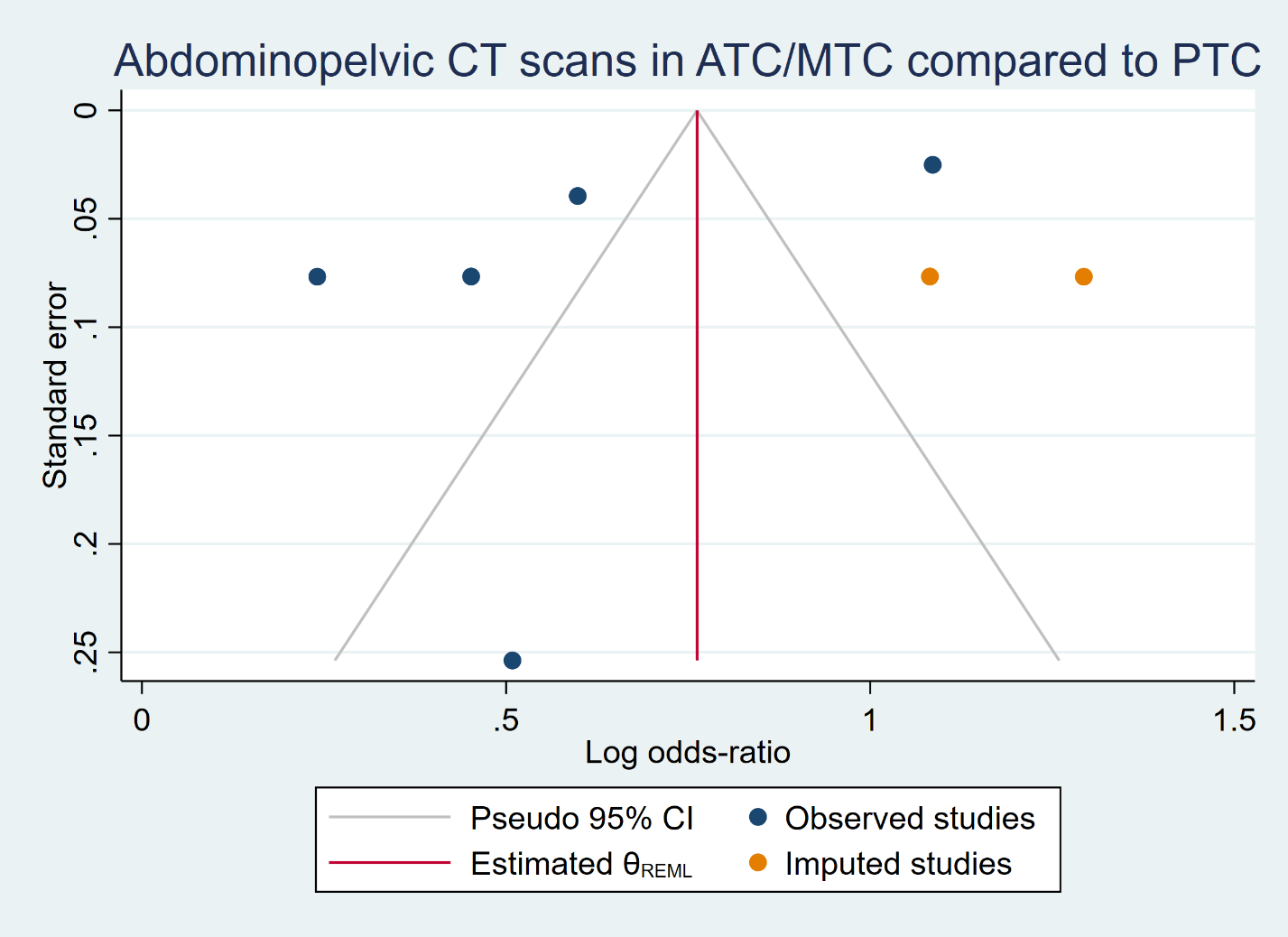


**Fig. 7.1** Funnel plot for assessing publication bias in the odds ratio meta-analysis of studies comparing rates of abdominopelvic CT scans in Adult or Mixed Trauma Centers with Pediatric Trauma Centers along with imputed studies based on nonparametric trim-and-fill analysis to address for publication bias. CI: Confidence Interval


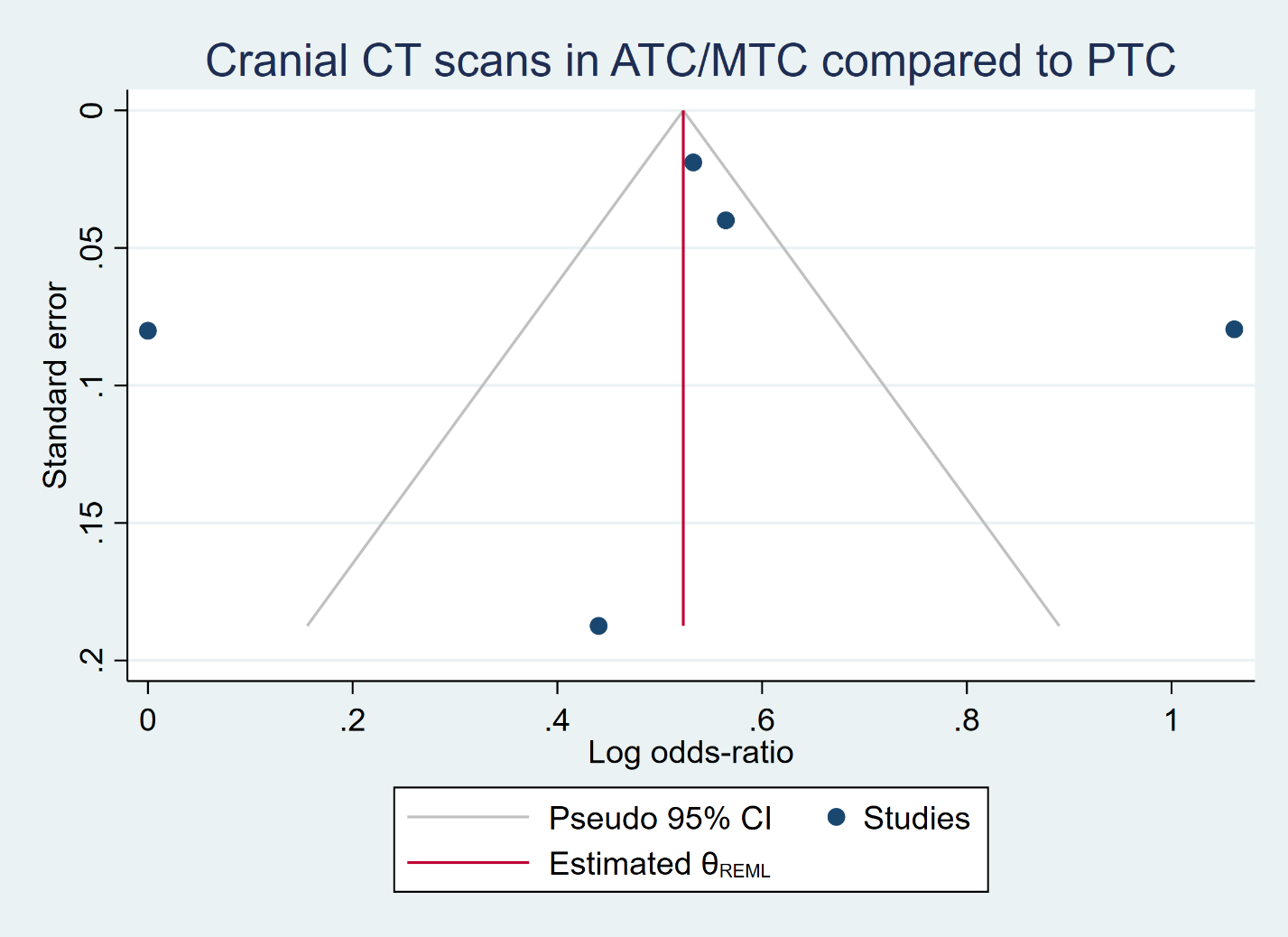


**Fig. 7.2** Funnel plot for assessing publication bias in the odds ratio meta-analysis of studies comparing rates of cranial CT scans in Adult or Mixed Trauma Centers with Pediatric Trauma Centers. CI: Confidence Interval


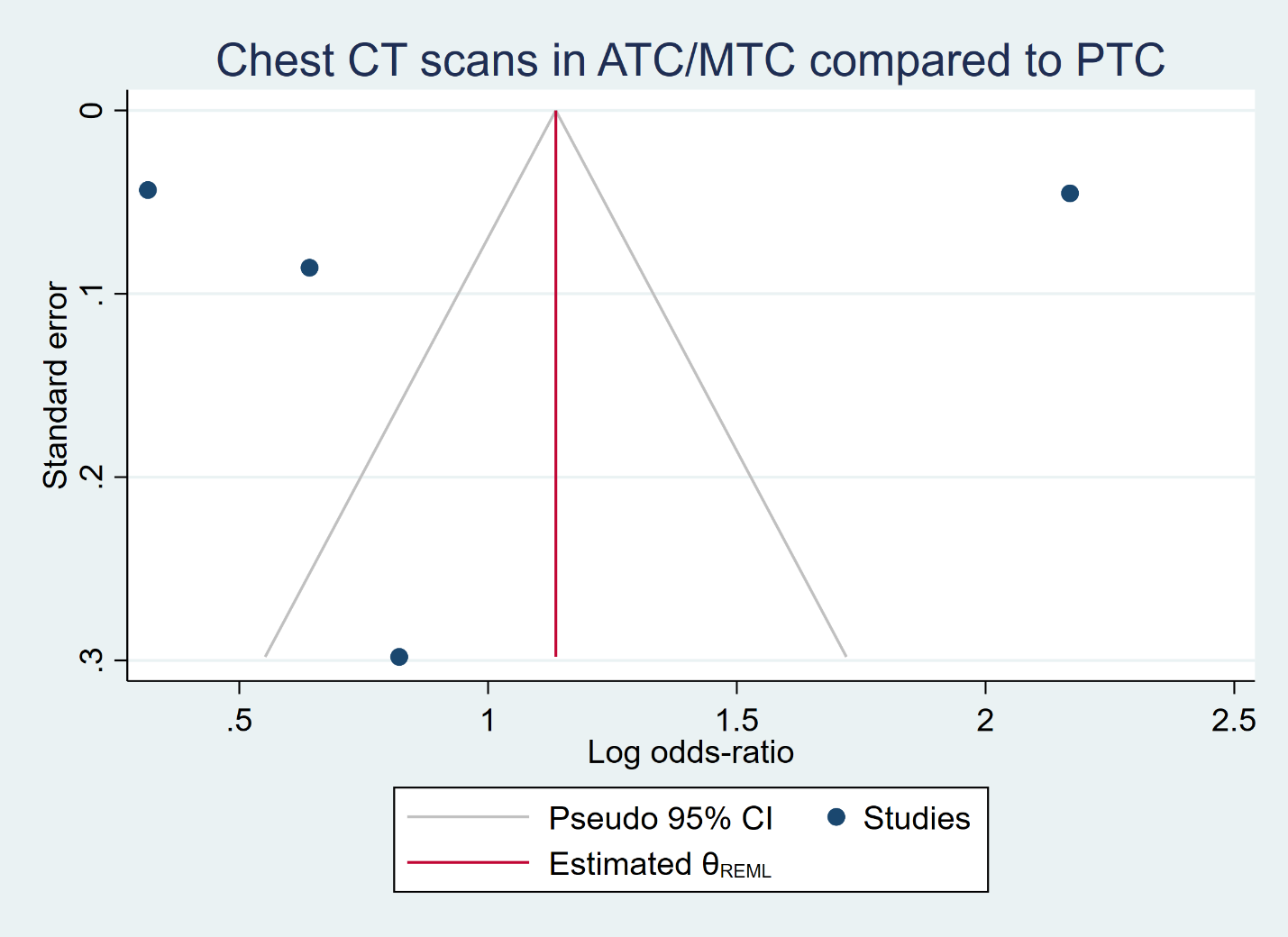


**Fig. 7.3** Funnel plot for assessing publication bias in the odds ratio meta-analysis of studies comparing rates of chest CT scans in Adult or Mixed Trauma Centers with Pediatric Trauma Centers. CI: Confidence Interval

| **Table 1.** The quality assessment of the included studies | | | | | | | | |
| --- | --- | --- | --- | --- | --- | --- | --- | --- |
| Study | Were the criteria for inclusion in the sample clearly  defined? | Were the study subjects and the setting described in  detail? | Was the exposure measured in a valid and reliable way? | Were objective, standard criteria used for  measurement of the condition? | Were confounding factors identified? | Were strategies to deal with confounding factors  stated? | Were the outcomes measured in a valid and reliable  way? | Was appropriate statistical analysis used? |
| Wiitala et al., 2022 (1) | Yes | Yes | Unclear | Yes | Yes | Yes | Yes | Yes |
| Walther et al., 2016 (2) | Yes | Yes | Unclear | Yes | Yes | Unclear | Yes | Yes |
| Walther et al., 2014 (3) | Yes | Yes | Unclear | Yes | Yes | Yes | Yes | Yes |
| Brinke et al., 2021 (4) * | Yes | Yes | Unclear | Yes | No | Unclear | Yes | Unclear |
| Streck et al., 2012 (6) | No | Yes | Unclear | Yes | No | Unclear | Yes | Yes |
| Stephens et al., 2017 (40) * | Yes | Yes | Yes | Yes | Yes | Yes | Yes | Yes |
| Sharma et al., 2022 (28) | Yes | Yes | Unclear | Yes | No | No | Yes | Yes |
| Schonfeld et al., 2013 (41) | Yes | Yes | Unclear | Yes | Yes | Yes | Yes | Yes |
| Sathya et al., 2018 (10) | Yes | Yes | Unclear | Yes | Yes | Yes | Yes | Yes |
| Plackett et al., 2015 (42) | Yes | Yes | Unclear | Yes | Yes | Unclear | Unclear | Yes |
| Phillips et al., 2021 (11) * | Yes | Yes | Unclear | Yes | Yes | Yes | Yes | Yes |
| Pariaszevski et al., 2022 (43) | Yes | Yes | Unclear | Yes | No | No | Unclear | Yes |
| Odia et al., 2020 (44) | Yes | Yes | Unclear | Yes | Yes | Unclear | Unclear | Yes |
| Nigrovic et al., 2015 (45) | Yes | Yes | Unclear | Yes | No | No | Yes | Yes |
| Moore et al., 2013 (46) | Yes | Yes | Unclear | Yes | Yes | Unclear | Unclear | Unclear |
| McGrew et al., 2018 (47) | Yes | Yes | Unclear | Yes | Yes | Unclear | Unclear | Yes |
| Mahdi et al., 2023 (48) | Yes | Yes | Unclear | Yes | Yes | Unclear | Unclear | Yes |
| Schonenberg Llach et al., 2021 (49) | Yes | Yes | Unclear | Yes | No | Unclear | Unclear | Yes |
| Livingston et al., 2013 (50) * | Yes | Yes | Yes | Yes | Yes | Unclear | Yes | Yes |
| Kuas et al., 2022 (51) | Yes | Yes | Unclear | Yes | Yes | Unclear | Yes | Yes |
| Kolousek et al., 2023 (52) | Yes | Yes | Unclear | Yes | Yes | No | Yes | Yes |
| Kim et al., 2005 (53) | Yes | Yes | Yes | No | No | No | Yes | Yes |
| Holmes et al., 2017 (54) | Yes | Yes | Yes | Yes | Yes | Yes | Yes | Yes |
| Haasz et al., 2015 (55) * | Yes | Yes | Unclear | No | Yes | Yes | Yes | Yes |
| Golden et al., 2016 (56) * | Yes | Yes | Unclear | Yes | Yes | No | Unclear | Yes |
| Gaffley et al., 2020 (57) | Yes | Yes | Unclear | Yes | Yes | Unclear | Yes | Yes |
| Edwards et al., 2021 (58) | Yes | Yes | Yes | Yes | Yes | Yes | Yes | Yes |
| Downie et al., 2023 (59) * | Yes | Yes | Unclear | Yes | No | Unclear | Unclear | Yes |
| Brinkman et al., 2015 (60) | Yes | Yes | Unclear | Yes | Yes | Unclear | Unclear | Unclear |
| Beno et al., 2022 (61) | Yes | Yes | Unclear | No | No | Unclear | Yes | Yes |
| * Exclusion from the meta-analysis occurred as a result of restricting the study sample to individuals with a high clinical suspicion, thus limiting their representation in the overall analysis | | | | | | | | |
